# Partial substitution in the hidden curriculum of children’s media: a comparative corpus analysis of religion-oriented and general-educational Turkish public broadcasting

**DOI:** 10.3389/fpsyg.2026.1887219

**Published:** 2026-08-25

**Authors:** Ramazan Şimşek

**Affiliations:** Department of Turkish Language and Social Sciences Education, Faculty of Education, Nevsehir Haci Bektas Veli Universitesi Egitim Fakultesi, Nevşehir, Türkiye

**Keywords:** CASEL, children’s broadcasting, comparative corpus analysis, educational media, hidden curriculum, Schwartz value theory, self-regulation, social–emotional learning

## Abstract

Research on social–emotional learning (SEL) has accumulated extensive evidence on programmatic outcomes but devoted less attention to the lexical-semantic shape of the educational input that children actually encounter. This study asks how religion-oriented and general-educational children’s broadcasting operationalize SEL competencies at the level of vocabulary. Two Turkish public children’s broadcasters, TRT Çocuk and TRT Diyanet Çocuk, were compared. The two share institutional auspices, regulatory oversight, target age range and broadcast format, yet differ systematically in curricular orientation (general-educational vs. religion-oriented). A 225,451-token comparable corpus (112,983 + 112,468; 24 series, 284 episodes) was analyzed using standard corpus-linguistic measures over morphologically lemmatized Turkish text: Simple Maths keyness, Gries’s deviation of proportions for dispersion, and collocation within a ± 5 token window. Pre-registered Mann–Whitney *U* tests with Bonferroni-Holm correction served as convergence indicators. The five CASEL competencies appeared in both channels at comparable rates (all SM ≤ 1.31; all DP < 0.18). Schwartz value axes diverged: tradition (SM = 2.50) and benevolence indexed the religion-oriented profile, whereas achievement (SM = 1.37) marked the general-educational channel. The most pronounced pattern emerged in self-regulation. Religious self-regulation appeared at SM = 7.94 in TRT Diyanet Çocuk (G^2^ = 656.2; Holm-corrected *p* < 0.001; rank-biserial |r| = 0.96). Secular self-regulation showed only a modest TRT Çocuk lead that did not reach inferential significance (SM = 1.56, p_adj = 0.423, null preserved). A theoretical concept of partial substitution is proposed to capture a pattern that neither a supplementation nor an outright displacement account describes: the religious repertoire gains dominance without fully crowding out secular techniques. Findings contribute to the input-characterization line in educational psychology with Turkish empirical material and offer implications for culturally responsive SEL curriculum design.

## 1 Introduction

Research on social–emotional learning (SEL) has, over three decades, become one of the fastest-expanding lines of inquiry in educational psychology (Saroglou, 2011). Durlak et al. (2011) meta-analysis of 213 intervention studies established that SEL programs produce statistically reliable gains in academic achievement, social behavior, and emotional competence. Taylor et al. (2017) followed students for up to 18 years and found these gains persist; the more recent meta-analysis by Cipriano et al. (2023) shows that SEL programs hold up even in post-pandemic classroom conditions. The five CASEL competencies (self-awareness, self-management, social awareness, relationship skills, and responsible decision-making) have settled into the field as the operational scheme for this research (Mahoney et al., 2018).

What the literature has paid less attention to is the input side. The lexical and semantic resources that surround children during development have not been mapped in systematic detail. This omission is conspicuous in the case of educational media. Educational psychology has a long line of input research, from Hart and Risley (1995) on early vocabulary exposure to Rowe (2012) on interaction-based input and Hirsh-Pasek et al. (2015) on input quality; each of these lines treats systematic quantification of language exposure as a precondition for any developmental claim. The educational-media side has produced strong companions, including Linebarger and Vaala (2010), Mares and Pan (2013) Sesame Street meta-analysis, and Fisch (2014), which converge on the point that direct content analysis is a prerequisite for any claim about viewer effects.

The well-developed line of work just sketched leans heavily on Western-centric content streams and secular content scheme. How SEL’s hidden curriculum (Jackson, 1968; Apple, 1979) is actually operationalized at the level of cultural-pedagogical repertoires, and how that operationalization shifts across cultural settings, has not been studied in proportionate detail. King and Boyatzis (2015) point out that the integration of children’s religious development with mainstream developmental psychology still awaits empirical material. Saroglou (2011) supplementation thesis — that religious development cannot be cleanly separated from secular psychology — leaves open what happens at the level of content: whether the two repertoires coexist in full, or whether the dominance of one contracts the visibility of the other. How religion-oriented and general-educational content actually operationalize SEL competencies in practice remains unexamined.

Turkey provides a setting in which the question can be examined with empirical traction. Two distinct children’s broadcasters operate within the Turkish Radio and Television Corporation (TRT): TRT Çocuk and TRT Diyanet Çocuk. Both function under the same institutional umbrella, the same regulatory body (RTÜK), the same language (Turkish), and the same target age range (3–10). The single dimension along which they diverge is curricular orientation. TRT Çocuk produces general-educational content; TRT Diyanet Çocuk produces religion-oriented content. The configuration yields a natural comparison case for the comparable-corpora approach (McEnery and Hardie, 2012), with curricular orientation isolated as the only varying design dimension.

The institutional-historical background of this dual-channel arrangement warrants a brief note. Religious content has expanded substantially in the Turkish educational system over the past two decades. Compulsory Religious Culture and Moral Knowledge (DKAB) instruction has been part of the curriculum since primary school; elective religious education courses were broadened after 2012; and the share of İmam Hatip schools rose throughout the 2010s (Çitak, 2013; Akşit et al., 2012). During the same period the Ministry of National Education systematized values-education programs and the Hayat Bilgisi (Life Studies) curriculum was revised in line with SEL-compatible competencies (MEB, 2018). TRT Diyanet Çocuk was established in 2015, in cooperation with the Presidency of Religious Affairs (Diyanet), and operates in parallel to mainstream TRT Çocuk broadcasting. This study makes no evaluative claim about that institutional context; it maps the linguistic-content profiles of the two broadcasting policies as descriptive data.

Building on this setting, the present study addresses four research questions. RQ1: At what rates and in what profile do the two broadcasting policies invoke the five CASEL competencies? RQ2: How do the two hidden curricula differ along the ten Schwartz value categories? RQ3: What pattern do self-regulation strategies (religious and secular repertoires) show across the two channels? RQ4: What additional content-differentiation patterns emerge from bottom-up keyness and collocation analyses beyond the theory-driven indicator pools?

The study claims to contribute on three levels. Empirically, the analysis provides the first systematic comparative corpus characterization of the SEL hidden curriculum profile across the two broadcasting policies of Turkish public children’s television. Theoretically, the question left open by Saroglou (2011) supplementation account — whether adding a religious repertoire leaves the secular one intact — is reframed under the partial substitution concept. Methodologically, a transparent analytical pipeline was followed in order to show how comparative corpus analysis can be positioned and examined as a replicable method for input characterisation in educational psychology, using corpus tools such as Sketch Engine, WordSmith Tools and AntConc.

One interpretive boundary must be flagged at the outset. The findings will show that, at the CASEL level, both channels invoke SEL targets at comparable rates. Read on its own, this surface similarity invites a naive equivalence reading. The analytic scheme of the study is designed to head off that drift: comparable SEL target structure plus differentiated operational repertoire. No evaluative judgment between the two content streams is offered. The contribution is a quantitative description of the lexical-semantic repertoires of both content streams.

## 2 Theoretical background

### 2.1 Social–emotional learning and the CASEL competencies

The CASEL scheme conceptualizes SEL around five interlinked competencies: self-awareness (recognition of emotions and values), self-management (emotion regulation, impulse control, stress management), social awareness (empathy, perspective taking), relationship skills (communication, cooperation, conflict resolution), and responsible decision-making (ethical reasoning, consequence appraisal). Durlak et al. (2011) meta-analysis documented that school-based SEL programs yield roughly an 11-percentile gain in academic achievement. These gains held up in Taylor et al. (2017) 18-year follow-up. Greenberg et al. (2017) examined the neurodevelopmental underpinnings linking SEL to cognitive executive function; Cipriano et al. (2023) report a standardized mean d ≈ 0.32 across 424 intervention studies in their recent meta-analysis.

Turkish-language SEL measurement instruments are still relatively new. Totan (2018) developed a Turkish social and emotional learning scale for adolescents. The relation between educational media and SEL has received some attention in the Turkish literature, but a comparative corpus-level analysis of the two channels examined here has not yet been carried out.

### 2.2 Schwartz’s theory of basic values

Schwartz (1992, 2012) identified ten basic values arranged along two conflicting higher-order axes: conservation (tradition, conformity, security) versus openness to change (self-direction, stimulation), and self-transcendence (benevolence, universalism) versus self-enhancement (achievement, power, hedonism). The scheme has been validated empirically in more than 80 countries (Schwartz and Boehnke, 2004; Schwartz et al., 2012). Demirutku and Sümer (2010) adapted the measure into Turkish and confirmed scale validity in adult samples.

Adapting Schwartz to media content analysis is feasible at the level of word frequency. Bardi and Schwartz (2003) showed the value-behavior link, and Knafo and Schwartz (2009) separated the family and media channels of value transmission. Whether the lexical signatures of values can be used for systematic comparison of children’s media content has, however, received comparatively little empirical attention.

### 2.3 Hidden curriculum

Jackson (1968) hidden curriculum names the normative, moral, and behavioral messages students systematically encounter without being directly taught. Apple (1979) drew out the ideological side of the hidden curriculum; Giroux (1983) located the concept within social reproduction theory. Within Bronfenbrenner and Morris (2006) bioecological systems theory, the microsystem inputs of children (family, school, media) carry the hidden curriculum. Educational television, especially in early childhood, is among the most systematically repeated sources of linguistic input that children encounter.

Research on the hidden curriculum in children’s media has been taken up by Anderson and Hanson (2017) on screen-based content and parent–child interaction. Linebarger and Walker (2005) documented the effects of educational programs on language development, and Kirkorian et al. (2008) mapped media effects at the cognitive-process level. A shared methodological boundary of these studies is that content analysis usually stops at categorical coding; systematic quantification of lexical microstructure is rarely carried out.

### 2.4 Religious and secular self-regulation

Religious self-regulation refers to the set of strategies through which religious frameworks structure the deferral, redirection, and reframing of impulse. McCullough and Willoughby (2009) model these as three goal-oriented processes — goal-setting, goal-pursuit, and goal-maintenance — mediated by patience as deferred action, gratitude as attentional redirection, and prayer as purpose-formation. Saroglou (2011) locates the behavioural dimension of religiosity within the “Big Four” paradigm, where it stands in close relation to self-regulatory processes.

The secular counterpart of the same construct draws on cognitive-behavioral intervention literature. Four core techniques recur across reviews of this tradition: diaphragmatic breathing, counting strategies, planned postponement, and mindfulness-based stress reduction. The operational definition of mindfulness was set out by Bishop et al. (2004); the meta-analytic evidence for mindfulness-based therapy on anxiety and depression was synthesized by Hofmann et al. (2010). Adaptations of these techniques for children, along with the supporting evidence base, are reviewed in Greenberg and Harris (2012).

The relation between the two repertoires has remained empirically open. Saroglou (2011) and Saroglou and Cohen (2011) defend a supplementation reading: religious values overlap with universal prosocial values and form an additional layer on top of secular strategies rather than displacing them. This reading carries an untested empirical commitment. If the religious repertoire is genuinely additive, secular strategies should remain as visible where it is culturally dominant as where it is not. The alternative — a displacement reading, under which cultural dominance diminishes the relative visibility of secular strategies — is equally consistent with the developmental literature and has not been ruled out. The two readings hinge on what cultural dominance does to lexical surface, and neither has yet been examined against comparable-corpora data. The present analysis takes up that empirical gap, and the resolution presented in section 5.2 occupies a position between the two camps rather than aligning with either.

### 2.5 Educational media content analysis

Content analysis of children’s educational media has followed two main lines. The first uses categorical coding: programs are sorted into content categories (educational, entertainment, advertisement) and compared on duration and frequency (Wilson, 2008; Calvert, 2015). The second focuses on effects analysis: relations between content features and viewer outcomes are tested with intervention or longitudinal designs (Mares and Pan, 2013; Fisch, 2014).

Comparative corpus-linguistic analysis of the linguistic-semantic microstructure of content streams has developed more slowly between these two main lines. Linebarger and Piotrowski (2009) examined the linguistic difficulty levels of children’s programs, but systematic comparison along SEL and value axes is rare. This gap is especially conspicuous in the case of religion-oriented children’s media. Roehlkepartain et al. (2006), in their critique of the methodological state of children’s religious development research, point to media content analysis as a place where systematization would help the field mature.

### 2.6 Comparable-corpus comparative content analysis

Corpus linguistics is methodologically kin to vocabulary input research in educational psychology (Hart and Risley, 1995), and adapting it to the educational-media setting is a natural step. Sinclair (1991) and Stubbs (2001) laid out the core principles of corpus-based analysis; Baker (2006) showed how to integrate corpus methods into critical discourse analysis. Brezina (2018) provides a systematic taxonomy of statistical measures in corpus linguistics. Kilgarriff (2005) thesis, “language is never, ever, ever, random,” questions the epistemic status of inferential statistics in corpus comparison and proposes effect-size-based descriptive reporting; that position shaped the analytic philosophy of this study.

Over the past decade, established corpus analysis software (Kilgarriff et al., 2014) has become the standard analytical infrastructure of corpus linguistics, backed by reference corpora available for more than 90 languages. WordSmith Tools, AntConc and Sketch Engine rank among the field’s most prominent instruments. Turkish, for its part, exhibits a rich inflectional morphology that a rule-based analyser resolves at the lemma level. The default measures within the platform are Simple Maths for keyword analysis (Kilgarriff, 2009), logDice and Mutual Information for collocational strength (Rychlý, 2008; Church and Hanks, 1990), and Gries (2008) DP for distributional dispersion. To preserve cross-study comparability, the analytical parameters of the present study were aligned with these defaults.

## 3 Method

### 3.1 Research design

This study is an input-characterization investigation that quantitatively maps the hidden curriculum dimension of educational media input. The design is positioned at the input level within the input-process-outcome chain of educational psychology; it does not aim at intervention or student-outcome measurement. The method maps the systematic differences between the linguistic-content profiles of the two channels in quantitative terms. Reception, causal effects, and evaluative judgments lie outside the scope of this study; these limits are discussed in §5.6 and 5.7.

### 3.2 Comparable corpora design

The study uses a comparable-corpora design for comparative content analysis (McEnery and Hardie, 2012). The two sub-corpora are matched on the following design dimensions: same institutional umbrella (TRT), same regulatory body (RTÜK), same language (Turkish), same target age range (3–10), and same broadcast format (animation and live-action educational programming). The single varying dimension is curricular orientation: TRT Çocuk produces general-educational content, TRT Diyanet Çocuk produces religion-oriented content.

The choice of channels is not incidental to the comparison. TRT Çocuk is the most-watched children’s television channel in Turkey (RTÜK, 2013), which makes its lexical repertoire the closest available proxy for the general-educational input that Turkish preschool and primary-age children actually receive; TRT Diyanet Çocuk is its religion-oriented public counterpart under the same broadcaster. Comparing the two therefore isolates curricular orientation while holding audience reach, institutional authority, and production norms broadly constant.

### 3.3 Corpus

Within each channel, the corpus comprises the originally produced series broadcast in the after-school to early-evening children’s band (approximately 16:00–21:00), the daypart of peak child viewership. Table 1 summarises the composition of the two sub-corpora.

**Table 1:** Corpus composition by channel.

Channel	Tokens	Episodes	Series
TRT Çocuk (general-oriented)	112,983	140	12
TRT Diyanet Çocuk (religion-oriented)	112,468	144	12
Total	225,451	284	24

The two sub-corpora are nearly perfectly matched in size (50.12%/49.88%). This balance structurally rules out the interpretive bias that asymmetric-corpus comparison can introduce in normalized frequency analysis (Gabrielatos and Marchi, 2011). Series-level token counts appear in Appendix B.

### 3.4 Transcription and preprocessing

The data were obtained from the channels’ official YouTube broadcasts. Automatic captions served as the starting layer; each episode was then watched in full for manual verification and correction. Inter-coder character-level agreement was 98.7%. Transcripts were stored in UTF-8 with Turkish-sensitive lowercasing (İ → i; I → ı) and non-letter characters removed. Sketch Engine and WordSmith Tools 7.0 were also drawn upon. Concordance-based context analyses, frequency counts and n-gram extraction were run through these programs in a controlled, stepwise manner.

### 3.5 Corpus analysis and measures

The corpus was analyzed with the standard corpus-analysis measures (Kilgarriff et al., 2014). Tokenization and lemmatization were carried out with a rule-based morphological analyzer for Turkish. Sketch Engine was likewise drawn upon. For each indicator pool, corpus lemmas were matched against theory-derived seed terms, and forms carrying privative or negation suffixes were excluded. The two sub-corpora (TRT Çocuk and TRT Diyanet Çocuk) were then compared using fpmw normalization, Simple Maths keyness, log-likelihood G^2^, logDice collocation, and Gries’s DP dispersion, all computed over lemmatized text.

The parameter set covers the following standard components: Turkish lemmatization with a rule-based morphological analyzer, fpmw normalization, Simple Maths keyness (N = 1; SM ∈ (1, 2) modest excess, (2, 5) moderate-pronounced excess, >5 large excess; Brezina, 2018), log-likelihood G^2^ as a convergence indicator (Dunning, 1993), lemma-based queries, and ±5 token window collocation analysis using logDice (Rychlý, 2008) and Mutual Information (Church and Hanks, 1990). Grammatical-relation-based word-sketch analysis was not used because a sufficiently strong Turkish dependency parser is not yet available. Interpretation guides for each measure appear in Appendix C.

### 3.6 Coding schemes and Indicator pools

The corpus was annotated according to two complementary theoretical schemes: the CASEL five-competency scheme (Durlak et al., 2011; Mahoney et al., 2018) and Schwartz’s value typology (Schwartz, 1992, 2012). Three additional thematic pools were created on top of these two: religious/spiritual vocabulary; religious self-regulation strategies (patience, gratitude, surrender, prayer); and secular self-regulation strategies (calming down, breathing, planning, focused attention). Trust-emotion and general emotion vocabulary pools were analyzed separately. A thematic summary appears in Table 2; full lemma lists and frequencies appear in Appendix A; interpretation guides for the measures appear in Appendix C.

**Table 2:** Thematic summary of the indicator pools.

Pool	Theme	Sample lemmas
CASEL: Self-awareness	Awareness of emotions/thoughts	feel, emotion, self, value, hope
CASEL: Self-management	Behavior regulation	patience, control, calm, discipline, plan
CASEL: Social awareness	Empathy, perspective	empathy, understanding, mercy, compassion, respect
CASEL: Relationship skills	Communication, cooperation	friend, share, team, peace
CASEL: Responsible decision	Ethical reasoning	responsible, right, wrong, morality, conscience, justice
Schwartz: Self-direction	Independent thought	free, independent, curiosity, creative, explore
Schwartz: Stimulation	Excitement, novelty	excitement, adventure, novelty, courage
Schwartz: Hedonism	Pleasure, fun	pleasure, enjoyment, fun, taste
Schwartz: Achievement	Ability, attainment	success, ability, reward, win, superior
Schwartz: Power	Authority, prestige	power, authority, leader
Schwartz: Security	Health, protection	trust, safety, protect, health, calm
Schwartz: Conformity	Obedience, respect for rules	obedience, harmony, rule, respect
Schwartz: Tradition	Cultural/religious tradition	tradition, custom, heritage, religion, faith, prayer
Schwartz: Benevolence	Compassion for close others	kindness, help, compassion, love, friendship, loyalty
Schwartz: Universalism	Justice, nature, equality	peace, equality, justice, tolerance, nature
Religious/spiritual vocabulary	Direct religious reference	allah, prophet, prayer, fasting, dua
Religious self-regulation	Religiously framed strategies	patience, gratitude, trust-in-God, prayer
Secular self-regulation	Non-religious strategies	calm, breath, count, plan, concentrate
Trust emotion	Trust, certainty, hope	trust, certain, believe, hope, relaxed
General emotion vocabulary	SEL-grounded emotion lexicon	happy, sad, afraid, anger, anxiety, pride

The pools were built from the core terms of the CASEL and Schwartz scales through morphological expansion against TS Corpus reference (Aksan et al., 2012) and ±5 token KWIC verification; uses that did not match the target sense, along with negation-suffixed derivatives, were excluded from matching. Two independent coders annotated the lemma pools: one a researcher with graduate training in educational psychology, the other a professor of language education; a third senior researcher resolved disagreements as adjudicator. Inter-coder agreement averaged Cohen’s *κ* = 0.86, with the highest reliability obtained for the religious-vocabulary pool (κ = 0.94) and the lowest for the CASEL self-management pool (κ = 0.78), where the boundary with the secular self-regulation pool — enacted technique versus characterological disposition — accounted for most disagreements. Lemmas reported in the appendix tables follow standard corpus-linguistic notation: full Turkish word forms appear without modification (e.g., merak, dua, sabır), while morphological root clusters that collapse multiple inflected forms appear with a trailing hyphen (e.g., keşf- covering keşif, keşfetmek, keşfedici; öğren−/öğret- covering öğrenmek, öğretmen, öğretim).

Three primary sources support the construct validity of the item pools. First, the core lemmas in the CASEL and Schwartz pools were drawn from items in psychometric instruments that have undergone Turkish validation studies: Demirutku and Sümer (2010) study on the Turkish adaptation of the PVQ-21 and Totan’s (2018) Turkish social and emotional learning scale for adolescents. The pool’s terms are therefore grounded in constructs whose scale-level validity has been independently established. Second, the morphological extension procedure did not alter the semantic content of the terms. Candidate derivatives were added to the list only after a Key Word in Context (KWIC) analysis confirmed their semantic congruence with the target construct. Polysemous words and forms carrying a negative suffix were consistently excluded throughout the procedure. Third, multi-source convergence at the content level is demonstrated by the alignment of three different analysis layers: theory-driven indicator pools, theory-independent inductive (bottom-up) keyword analysis, and collocation profiles. This operational measurement procedure should not be treated as an individual-level psychometric assessment. It functions as an indicator of the lexical density of theory-based constructs in publicly available published content. In this case, the appropriate validity criterion is content-level convergence rather than factor structure at the scale level (Baker, 2006; Bednarek and Caple, 2017; Brezina, 2018).

### 3.7 Unit of analysis and metrics

The unit of analysis throughout this study is the individual TV series rather than the token or the episode. Series-level granularity preserves the interpretive value of Gries (2008) DP dispersion measure and removes the weighting bias that within-series variation in episode counts would otherwise introduce into normalized frequency comparisons. Each category receives five output values: raw frequency, fpmw, Simple Maths in both directions, log-likelihood G^2^ as a convergence indicator, and Gries DP as a dispersion check. DP values below 0.5 indicate channel-level distributed patterns; values at or above 0.5 indicate series-specific concentration. For target lemmas, ±5 token collocation analysis (logDice and MI) was added as a supplementary layer. Numerical comparisons throughout were verified against KWIC inspection within a ± 6 token window. Interpretation guides for each measure are reproduced in Appendix C.

### 3.8 Bottom-up Keyness analysis

Data-driven keyness analysis was carried out in two layers. The first layer is channel signature analysis: Simple Maths ranking is computed over all filtered lemmas. Because this list structurally includes character and program names, names of characters, names of series/programs, YouTube-platform terms, exclamations (hey), and series-specific noise terms (inci) were excluded from the analysis. This filter is a standard noise-cleaning step in corpus-based content analysis (Baker, 2006; Brezina, 2018). Religious person/place names (Allah, prophet, Kaaba, Zamzam) were retained as content markers. The second layer is content-differentiation analysis: Simple Maths and G^2^ rankings among lemmas with at least 3 occurrences in both channels, which removes mechanical single-channel asymmetry.

### 3.9 Prior commitment and statistical convergence

Hypotheses and the analysis plan were committed to in advance of data extraction. Descriptive corpus analysis serves as the primary evidence base of this study, a position grounded in Kilgarriff (2005) argument that *p*-values in closed-corpus comparisons cannot bear a stand-alone inferential burden. Mann–Whitney *U* tests with Bonferroni-Holm correction were applied as convergence indicators in support of the descriptive analysis rather than as the primary statistical claim. Full inferential results are reported in Appendix D.

Because the unit of analysis is the series (*n* = 12 per channel), the inferential layer is powered to detect only large between-channel effects. For a two-sided Mann–Whitney *U* test with *n* = 12 per group at power 0.80 and *α* = 0.05, the minimum detectable effect is approximately |*r*| = 0.62, rising to roughly 0.67 to 0.73 under Bonferroni-Holm adjustment. The two significant contrasts sit well above this threshold (religious self-regulation, |*r*| = 0.96; religious vocabulary, |*r*| = 0.82), whereas the effect for secular self-regulation (|*r*| = 0.36) falls below it. The design is therefore adequately powered for the large lexical asymmetries on which the substantive claims rest, but not for a modest effect of the secular self-regulation magnitude, which is accordingly interpreted descriptively rather than as evidence of equivalence.

### 3.10 Ethics

The study involves no human or animal participants and falls outside the scope of institutional ethics committee approval. Methodological limitations are discussed in detail in §5.6.

## 4 Findings and analysis

### 4.1 Corpus-level descriptive statistics

The two sub-corpora are nearly identical in size: 112,983 tokens in TRT Çocuk versus 112,468 in TRT Diyanet Çocuk, a difference of 0.46%. Total episode counts come to 140 and 144 respectively, distributed across 12 series in each channel. Within-series token counts range from 5,149 to 20,907 in TRT Çocuk (median 8,510) and from 3,325 to 17,022 in TRT Diyanet Çocuk (median 8,854). The ranges are comparable, and no individual series acts as a dominant outlier within either subcorpus. This near-balance constitutes a design-level safeguard against the interpretive bias that asymmetric-corpus comparison can introduce into normalized frequency analysis (Gabrielatos and Marchi, 2011); the property is built into the comparable-corpora design rather than achieved through post-hoc adjustment.

### 4.2 Schwartz value categories

**Figure 1:**
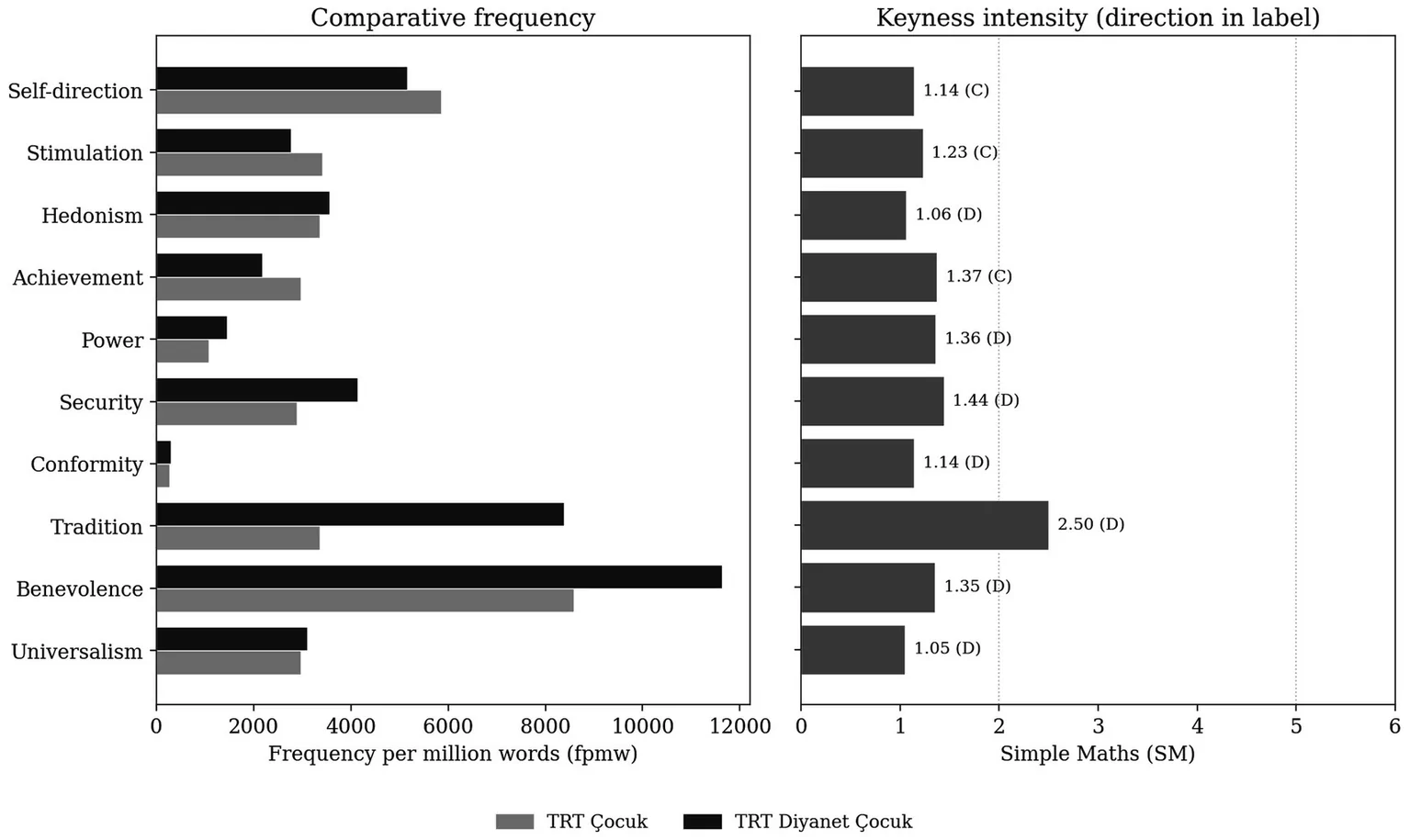
Comparative profile of the ten Schwartz value categories (fpmw and Simple Maths).

The lexical signatures of the ten Schwartz value categories are summarized in Table 3. For visualization, see Figure 1.

**Table 3:** Corpus metrics for the Schwartz value categories.

Schwartz value	Çocuk fpmw	Diyanet fpmw	SM	Direction	G^2^	DP
Tradition	3,354	8,376	2.50	Diyanet>	250.4	0.248
Security	2,885	4,143	1.44	Diyanet>	25.5	0.230
Hedonism	3,354	3,566	1.06	Diyanet>	0.7	0.227
Benevolence	8,585	11,630	1.35	Diyanet>	51.9	0.165
Power	1,071	1,458	1.36	Diyanet>	6.7	0.428
Universalism	2,965	3,103	1.05	Diyanet>	0.4	0.294
Conformity	266	302	1.14	Diyanet>	0.3	0.405
Self-direction	5,859	5,157	1.14	Çocuk>	5.0	0.124
Stimulation	3,408	2,765	1.23	Çocuk>	7.5	0.201
Achievement	2,974	2,178	1.37	Çocuk>	13.9	0.177

Two distinct use patterns emerge from Table 3. On TRT Diyanet, terms from the tradition pool occur roughly 2.5 times more frequently than on TRT Çocuk (8,376 vs. 3,354 fpmw), a difference that is highly significant (G^2^ = 250). This lexical concentration is expected, since the tradition pool encompasses both cultural and religious components. The pattern supports placing TRT Diyanet within Schwartz (2012) higher-order conservation category.

The opposite picture appears in the achievement category. Achievement-related terms occur 1.4 times more frequently on TRT Çocuk than on TRT Diyanet (2,974 vs. 2,178 fpmw; G^2^ = 14). The two channels thus align with opposing axes in Schwartz’s higher-order value structure: TRT Çocuk with self-enhancement (achievement), TRT Diyanet with conservation and self-transcendence (tradition and benevolence). Knafo and Schwartz (2009) predicted that the value transmission characteristics of media channels can be identified at the word level, and the present findings offer empirical support for this hypothesis in the context of instructional media.

Analytically, however, the benevolence dimension shows an unbalanced pattern. Descriptively, TRT Diyanet shows the higher frequency (11,630 vs. 8,585 fpmw; SM = 1.35, G^2^ = 51.9). Under the Holm correction, the series-level Mann–Whitney test does not reach statistical significance (*p* = 0.470). We report this gap between descriptive and inferential statistics openly rather than setting it aside. In absolute terms, both channels make extensive use of benevolence-related expressions. When absolute frequency is this high, the resulting proportional differences do not necessarily translate into systematic variation in the series-level distributions.

The power category sits at DP = 0.428, close to the conventional 0.5 threshold for series-specificity. Some component of the observed lean is therefore likely to derive from particular series rather than from a channel-level pattern, and interpretation should accommodate that nuance. Overall, the Schwartz profile supports the placement of TRT Diyanet in conservation plus self-transcendence and TRT Çocuk in self-enhancement. The remaining categories all display distribution at the channel level (DP < 0.5), with power as the borderline exception.

### 4.3 Composition of self-regulation strategies: the central finding

**Figure 2:**
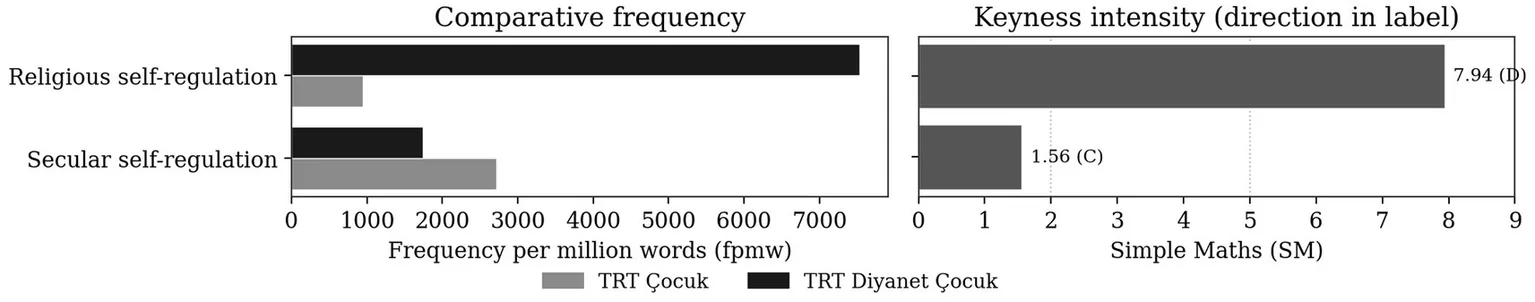
Counter-complementary profile of self-regulation repertoires (central finding).

The central finding of the study is the counter-complementary distribution of self-regulation strategies. The finding is summarized in Table 4 and visualized in Figure 2.

**Table 4:** Self-regulation repertoires.

Category	Çocuk fpmw	Diyanet fpmw	SM	Direction	G^2^	DP
Religious self-regulation	947	7,531	7.94	Diyanet>	656.2	0.439
Secular self-regulation	2,717	1,743	1.56	Çocuk>	24.2	0.187

The table records the most pronounced differentiation between the two channels. Religious self-regulation is invoked roughly 8 times more often in TRT Diyanet (7,531 vs. 947 fpmw). The lean falls into the “large excess” magnitude at SM = 7.94; the G^2^ value of 656.2 confirms the exceptional magnitude of the difference. The Mann–Whitney *U* convergence test supports the finding with Holm-corrected *p* < 0.001 and rank-biserial |*r*| = 0.96 (Appendix D). Secular self-regulation runs in the opposite direction: about 1.6 times more often in TRT Çocuk (2,717 vs. 1,743 fpmw), at SM = 1.56 in the “modest excess” magnitude. In the Mann–Whitney test this lean did not reach inferential significance (Holm-corrected *p* = 0.423); the null hypothesis was preserved. The religious repertoire appears with clear dominance within TRT Diyanet, while the secular repertoire shows only a slight lean within TRT Çocuk. This counter-complementary distribution is termed partial substitution throughout the analysis (developed further in §5.2).

This result directly aligns with the religious self-regulation model proposed by McCullough and Willoughby (2009). The model they created states that three goal-oriented processes (goal-setting, goal-pursuit, and goal-maintenance) are how religiosity functions. These processes are mediated by three essential components: “patience” as deferred action, “gratitude,” which helps redirect attention, and “prayer,” which shapes purpose. Throughout the corpus, TRT Diyanet Children’s material exhibits these processes with a high lexical density. Religion-focused programs systematically include the “behavior” dimension, which is part of Saroglou (2011) “Big Four” paradigm and is closely related to self-regulation processes. However, TRT Diyanet does not completely lack the secular (nonreligious) self-regulation repertoire operationalized by Bishop et al. (2004) and Hofmann et al. (2010). This repertoire, which includes techniques like diaphragmatic breathing, counting, and deliberate delay, is still comparatively underrepresented, but its existence in the corpus has been shown empirically.

Key word in context (KWIC) analysis makes this distribution’s qualitative texture even more apparent. On TRT Diyanet self-regulatory discourses are always positioned within a religious context. the lemma “patience,” for instance is frequently used in phrases like “O Lord grant me patience so that I may recover soon,” “O Allah you help those who are patient,” and “there is ease in every difficulty; be patient.” a similar structure is also used to communicate the idea of gratitude: “O Lord praise be to you,” “praise be to Allah my health is fine.” conversely statements like “put your trust in Allah my child” and “I have placed my trust in my Lord” convey submission or faith in god. The TRT Çocuk channel also uses the same lemmas although they are essentially employed in rather different situations. Sentences like “be a little patient; this balloon will surely inflate” and “let us be patient; let us pick the cherry ones first then the strawberry ones” are examples of patience on this channel. On the other hand expressions like “Oh thank goodness this job is finally done” and “thank goodness we left early; we avoided the traffic” are examples of common expressions of thankfulness. A significant difference at the pragmatic level is concealed by this apparent resemblance at the lemma level. this difference is investigated in further detail in section 4.7 using the collocation profile of the word “Allah.”

Consideration should also be given to a particular example at the series level. TRT Diyanet’s Mercanya series provides a clear example of secular self-regulation practices. A systematic breathing exercise scenario appears when the collocation profile of the lemma “nefes” within a ± 5-word span is investigated. Trusting (logDice = 12.85), taking (11.68), giving (11.33), and one’s own (11.18) are the key collocates in this profile. This systematic trend is also supported by Key Word in Context (KWIC) analysis. Concrete instances include statements such as “we are taking a deep breath now, exhaling slowly,” “we are breathing in, breathing out, I trust myself, I can do it,” and “we breathe in through our nose, out through our mouth, we are calming down.” Evidently, secular self-regulation strategies are not completely excluded by a channel that focuses on religion. Rather than a categorical rejection, the low overall frequency of these approaches indicates a reduction in their use. The descriptive picture in this study is labeled “partial substitution.”

### 4.4 CASEL competencies

**Figure 3:**
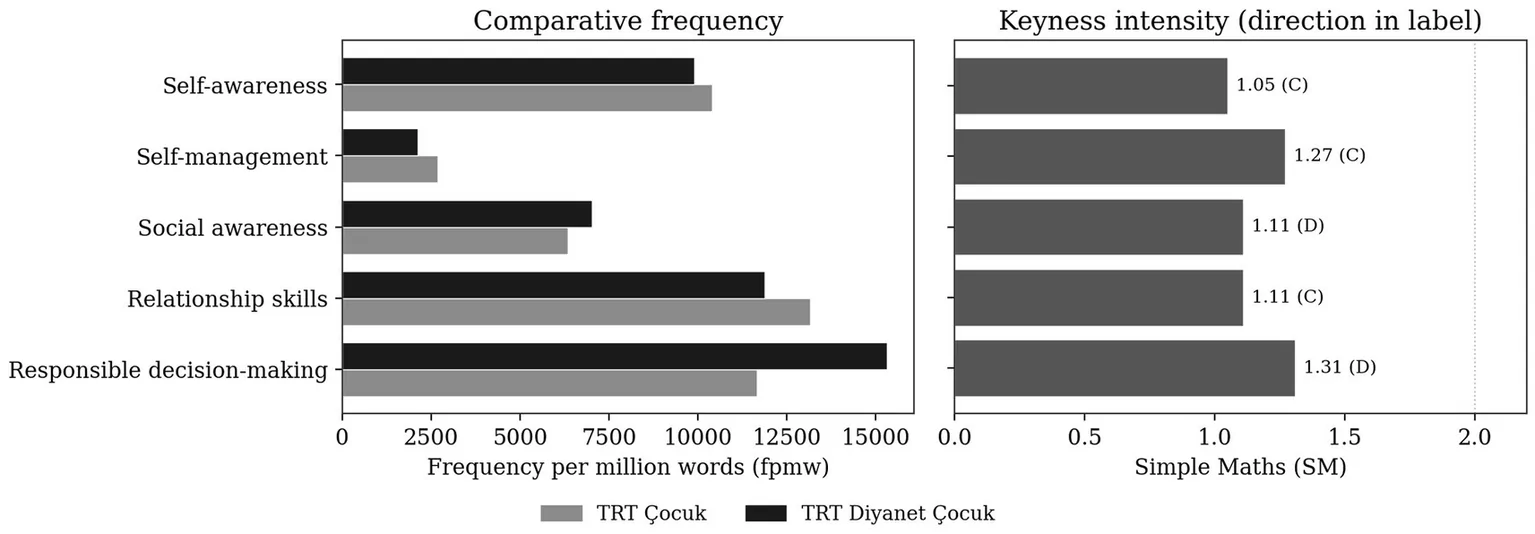
Comparative frequency profile of the five CASEL competencies.

The profile of the five CASEL competency categories is presented in Table 5 and Figure 3.

**Table 5:** The five CASEL competencies.

Competency	Çocuk fpmw	Diyanet fpmw	SM	Direction	G^2^	DP
Responsible decision-making	11,666	15,311	1.31	Diyanet>	55.7	0.161
Relationship skills	13,152	11,879	1.11	Çocuk>	7.3	0.114
Self-awareness	10,409	9,905	1.05	Çocuk>	1.4	0.128
Social awareness	6,337	7,015	1.11	Diyanet>	3.9	0.103
Self-management	2,691	2,125	1.27	Çocuk>	7.5	0.160

Table 5 demonstrates that each of the five CASEL competencies is invoked at substantial frequency in both channels. The categories vary in absolute density (responsible decision-making is most heavily represented at roughly 13,500 fpmw, self-management least heavily at around 2,400 fpmw), but the cross-channel margins of differentiation remain narrow throughout. All Simple Maths values fall at or below 1.31; no single competency is invoked more than 1.5 times as often in one channel relative to the other. Dispersion indices (DP) consistently fall below 0.18, indicating that each competency is broadly distributed across the channel rather than concentrated within one or two series. Both channels therefore display broad coverage of the CASEL scheme at the lexical surface level.

This finding carries notable descriptive weight for educational psychology. The five CASEL competency categories are structurally invoked in both broadcasting policies, present side by side at the lexical level. The difference between the two hidden curricula does not lie in the presence or absence of the CASEL categories. The difference lies in how each competency is operationalized below the surface. Within the same CASEL self-management category, one channel produces lexical items such as “calming down,” “planning,” and “impulse control”; the other fills the same category with “patience,” “gratitude,” “surrender,” and “prayer.” The categorical scaffold is shared; the operational content diverges substantially, a point taken up in Discussion §5.1 and §5.4.

### 4.5 Religious/spiritual vocabulary

Direct religious and spiritual vocabulary appears at 14,031 fpmw in TRT Diyanet Çocuk and at 4,372 fpmw in TRT Çocuk. The difference, with Simple Maths at 3.21 (in the moderate-to-pronounced range) and G^2^ at 601.3, constitutes one of the two most systematic channel-level differentiations observed across the analysis, alongside the self-regulation contrast reported in §4.3. The Mann–Whitney convergence test supports the finding with Holm-corrected *p* = 0.001 and rank-biserial effect size |*r*| = 0.82 (Appendix D). Dispersion at DP = 0.362 establishes that the finding is distributed across the channel rather than driven by particular outlier series.

**Figure 4:**
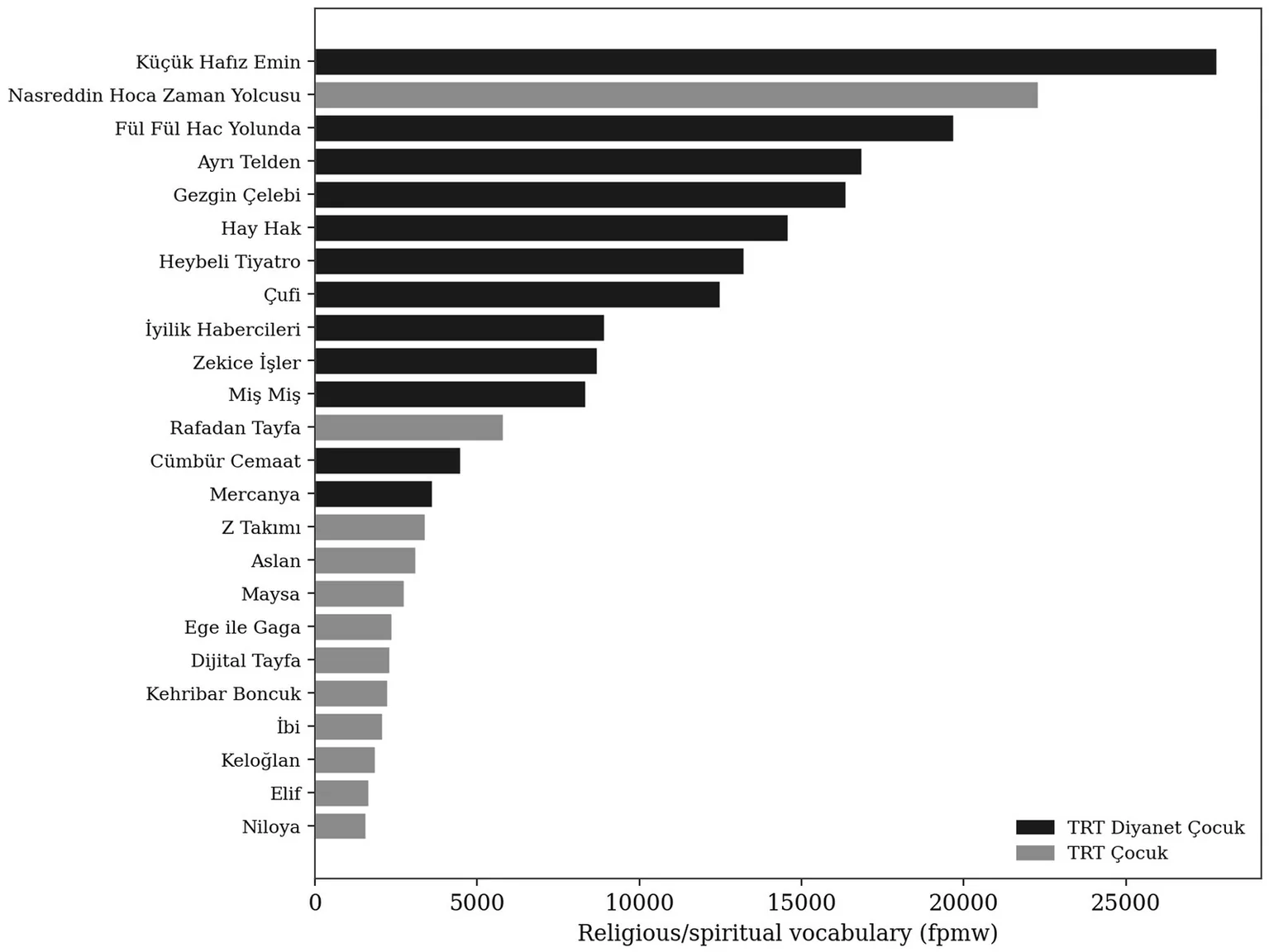
Series-level distribution of religious/spiritual vocabulary.

Series-level distribution is presented in Figure 4. Within TRT Diyanet, all series except the least religiously dense one (Mercanya, approximately 3,600 fpmw) fall above the TRT Çocuk channel average of 4,372 fpmw. Within TRT Çocuk, the single exception to the channel pattern is Nasreddin Hoca Zaman Yolcusu. The series centers on a cultural-religious folk figure of medieval Anatolian tradition, and the resulting density of religious vocabulary is structurally explicable rather than anomalous. This nuance suggests that the line between the two channels is best read as a graded intensity scheme rather than a categorical divide. The religious-cultural substrate is not entirely excluded from the secular-oriented channel; its density there is simply substantially lower.

### 4.6 Content-differentiation key lemmas

**Figure 5:**
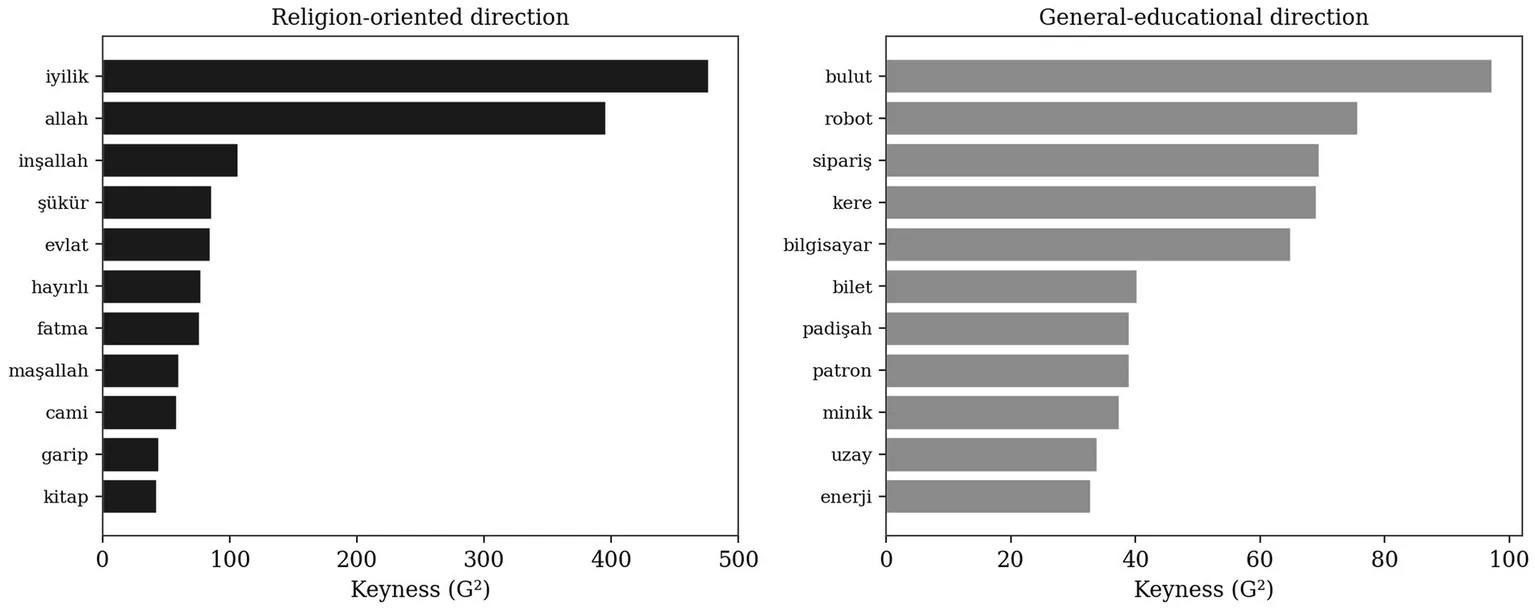
Content-differentiation key lemmas (proper nouns and noise terms filtered).

When character/program proper nouns and noise terms (including hey, inci) are filtered out, and the requirement of at least 3 occurrences in both channels is imposed, the key lemma list showing genuine content differentiation emerges (Table 6; Figure 5).

**Table 6:** Content-differentiation key lemmas (G^2^-ranked).

Diyanet direction	G^2^	SM	Çocuk direction	G^2^	SM
iyilik (goodness)	476.3	62.6	bulut (cloud)	97.2	18.3
allah	395.4	6.8	robot	75.6	5.2
inşallah	106.4	17.1	sipariş (order)	69.4	20.8
şükür (gratitude)	85.2	16.5	kere (times)	69.0	8.0
evlat (child)	84.5	10.6	bilgisayar (computer)	64.8	8.8
hayırlı (auspicious)	76.9	18.1	bilet (ticket)	40.2	13.5
fatma (Fatma)	75.9	11.7	padişah (sultan)	38.9	13.2
maşallah	59.6	18.4	patron (boss)	38.9	13.2
cami (mosque)	58.0	8.8	minik (tiny)	37.3	5.7
garip (strange)	43.7	4.0	uzay (space)	33.8	3.6
kitap (book)	42.2	2.3	enerji (energy)	32.8	9.5

The key lemma profiles in the two directions present two distinct thematic architectures. The Diyanet-direction lemmas cover religious vocabulary (allah, inşallah, gratitude, maşallah, auspicious, mosque) and religious-family references (child, Fatma). The Çocuk-direction lemmas show three lines: technology and modernity (robot, computer, space, energy), the classical fairy-tale repertoire (sultan), and everyday consumption and interaction (order, ticket). Character and program proper nouns (rafadan, kamil, niloya, mercanya, heybeli tiyatro, mavi characters), exclamations (hey, inci), and series-specific noise terms were removed from the analysis under the standard procedure described in Method §3.8. The two directions therefore trace a modernity–tradition contrast at the lexical surface, consistent with the divergence along the Schwartz value axes reported in Section 4.2.

### 4.7 Pragmatic divergence: collocate profiles of target lemmas

±5 token window collocation analysis enriches the frequency-based findings with the dimension of pragmatic use. This section first inspects the most pronounced case of pragmatic divergence in the study, the lemma “Allah,” and then documents the systematic character of the pattern through four additional target lemmas.

#### 4.7.1 The pragmatic split of “Allah”

Among all target lemmas examined, “Allah” displays the highest occurrence asymmetry between the two channels (81 occurrences in TRT Çocuk against 552 in TRT Diyanet) and, for the theoretical argument of the present study, the sharpest pragmatic divergence.

In TRT Çocuk, the strongest collocates (logDice) are: *anahtar* (key, 11.86), *hey* (10.55), *bahçe* (garden, 10.43), *kıl* (10.23), *hay* (9.97). This profile reflects the secularized everyday-use pattern of the “Allah Allah!” exclamation. KWIC contexts: *“hay Allah anahtarımı kaybettim yine”* (oh Allah, I lost my key again), *“Allah Allah ne tuhaf bu durum”* (Allah Allah, how strange this is), *“Allah aşkına biraz sessiz olun”* (for Allah’s sake, be quiet a moment), *“Allah’tan kimse görmedi yoksa rezil olurduk”* (thank Allah no one saw, otherwise we’d have been embarrassed). These patterns, settled into everyday Turkish, occur frequently without carrying actual religious reference; “Allah” works as a marker of surprise, mild rebuke, or light emphasis.

**Figure 6:**
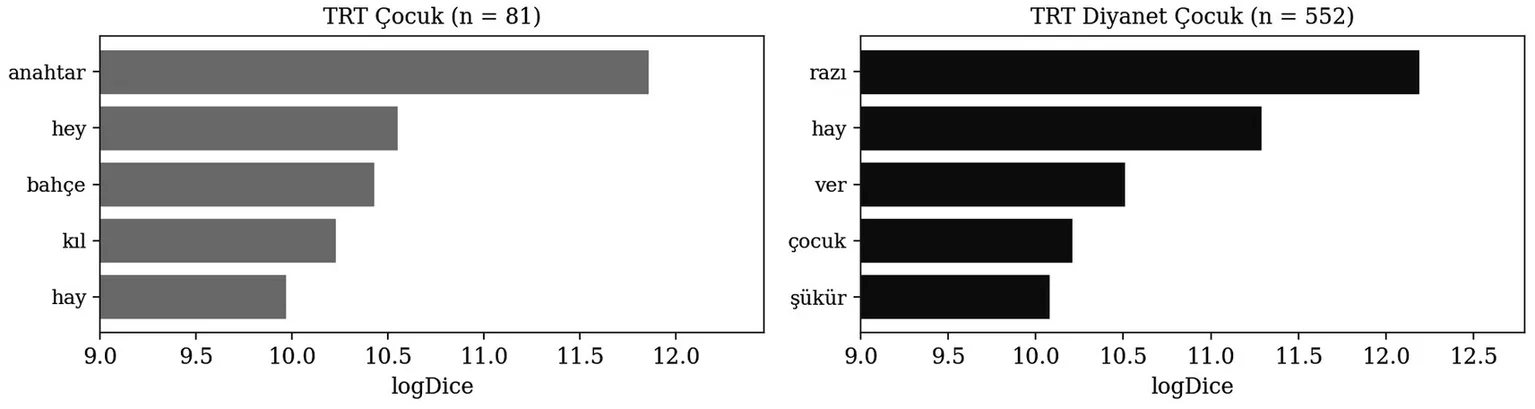
Pragmatic function asymmetry of “Allah” (±5 token collocation, logDice).

In TRT Diyanet, the collocate profile differs: *razı* (pleased, 12.19), *hay* (11.29), *ver* (give, 10.51), *çocuk* (child, 10.21), *şükür* (gratitude, 10.08). These collocates are direct indicators of religious approval, permission, and gratitude rhetoric. KWIC contexts: *“Allah razı olsun çok yardımcı oldun”* (may Allah be pleased with you, you helped a lot), *“Allah’ın izniyle başarırız bu işi”* (with Allah’s permission we’ll succeed), *“Allah’a şükür sağlığım yerinde”* (thanks to Allah, my health is good), *“Allah’ım niyet ettim senin rızan için”* (my Allah, I have set my intention for your pleasure), *“çocuklar Allah’ı seven onun sevdiklerini de sever”* (children, one who loves Allah also loves those whom Allah loves). Here the lemma carries direct theological reference (see Figure 6).

#### 4.7.2 Collocate profiles of other target lemmas

**Figure 7:**
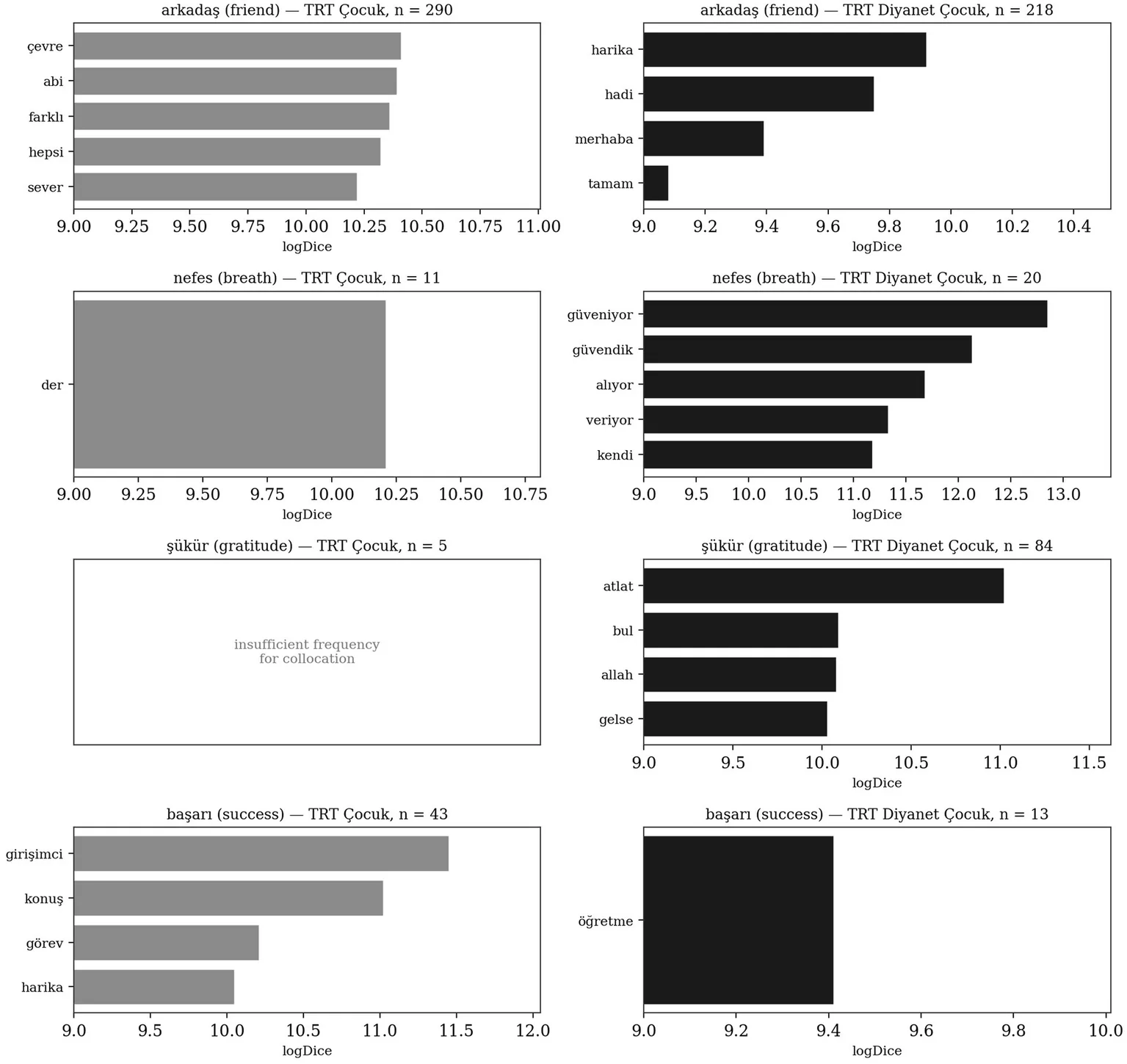
Channel-based collocate profiles for four target lemmas (±5 tokens, logDice).

To establish that the pragmatic-divergence pattern is not confined to the single case of “Allah,” four further target lemmas were analyzed (Figure 7): *arkadaş* (friend; social skill; dense in both channels), *nefes* (breath; secular self-regulation; modeled within Diyanet’s Mercanya), *şükür* (gratitude; religious self-regulation; predominant in Diyanet), and *başarı* (success; self-enhancement; predominant in Çocuk). Each lemma reveals a distinct manifestation of the underlying pragmatic-divergence pattern.

The *arkadaş* lemma occurs at high frequency in both channels (290 occurrences in TRT Çocuk against 218 in TRT Diyanet), yielding superficial similarity at the count level. The collocate profiles, however, point to substantially different pragmatic environments. TRT Çocuk produces collocates including çevre, abi, farklı, hepsi, and sever, which evoke an in-group interaction frame coupled with explicit difference-tolerance: *“farklı arkadaşlarımız hepsi aramızda yerini aldı”* (our different friends have all taken their place among us). TRT Diyanet produces a contrasting set (harika, hadi, merhaba, tamam) reflecting direct greeting and welcoming formulae: *“hadi arkadaşlar merhaba”* (come on friends, hello), *“tamam arkadaşlar haydi başlayalım”* (okay friends, let us start). Identical lemma at comparable frequency, settling into different discourse architectures.

The *nefes* lemma, occurring 20 times in TRT Diyanet, produces a particularly distinctive collocate profile: güveniyor (12.85), güvendik (12.13), alıyor (11.68), veriyor (11.33), kendi (11.18). The combination directly reproduces the structured breathing exercise from the Mercanya series: *“nefes alıyoruz nefes veriyoruz kendime güveniyorum başarabilirim”* (breathing in, breathing out, I trust myself, I can do it). TRT Çocuk yields only 11 occurrences and a single salient collocate (der, logDice = 10.21). The contrast makes the partial-substitution argument visible at the lemma level: TRT Diyanet does not exclude secular self-regulation techniques but uses them at substantially lower density.

The *şükür* lemma displays the most asymmetric distribution among the target set (5 vs. 84 occurrences). TRT Çocuk does not generate collocates at sufficient frequency for analysis. TRT Diyanet generates collocates atlat (11.02), bul (10.09), allah (10.08), gelse (10.03), all carrying prayer-based rhetoric of thankfulness: *“Allah’a şükür hastalığı atlattık”* (thank Allah, we got through the illness), *“şükür Allah’ım nimetine”* (thank you my Allah for your blessing). One note of complication: Turkish carries a secularized everyday use of “şükür” in the form *“of çok şükür”* (oh, thank goodness). Even that secular variant does not appear at sufficient density within TRT Çocuk. The compositional differentiation within the religious self-regulation repertoire established in §4.3 is independently confirmed at this lemma level.

The *başarı* lemma displays asymmetric distribution in the opposite direction (43 in TRT Çocuk against 13 in TRT Diyanet). Collocates within TRT Çocuk include girişimci (11.45), konuş (11.02), görev (10.21), and harika (10.05), evoking contexts of individual performance, presentation skill, and task achievement: *“genç girişimci sunumunda büyük başarı”* (young entrepreneur, great success in their presentation), *“harika konuşmayla görevi başardın”* (with a wonderful speech you succeeded in your task). TRT Diyanet generates a single salient collocate, öğretme (teaching, 9.41), placing the concept of success in a teacher-student context rather than an individual-performance one. The 1.37-fold over-representation of Schwartz achievement vocabulary in the Çocuk channel (§4.2) corresponds cleanly to this pragmatic profile.

Taken together, the five-lemma analysis brings out the systematic character of the “same surface word, different pragmatic function” pattern. Stubbs (2001), in his foundational work on semantic prosody, established the principle most clearly: a word’s meaning is built up through the lexical environment that surrounds it, and the surface form alone communicates only a fraction of pragmatic content. Frequency-based analysis cannot, in isolation, measure either religious or secular density. Two methodological implications follow for content analysis. First, raw frequency distribution may not fully reflect pragmatic meaning. Second, KWIC inspection together with collocation analysis must be treated as indispensable analytic layers rather than as optional supplementary checks.

## 5 Discussion

### 5.1 The lexical-semantic architecture of the two hidden curricula

The interpretation of the findings is organized in three hierarchical analytic layers. Each layer offers a different order of information, and the relation across layers carries the central theoretical contribution of the study.

The first layer is descriptive. The five CASEL competency categories are broadly distributed across both channels (all DP < 0.18, all SM ≤ 1.31). The two broadcasting policies invoke the same SEL target categories at comparable rates. This descriptive convergence supports the conclusion that religion-oriented and general-educational children’s content share a common SEL target structure at the categorical level. The five competencies defined by the CASEL scheme are observable in the shared lexical architecture of both content streams. The finding provides empirical support for the cross-cultural universality of the SEL category structure that Cipriano et al. (2023) proposed in their recent meta-analytic synthesis.

The second layer is analytic. The Schwartz value axes examined in §4.2 and the self-regulation repertoires examined in §4.3 disclose systematic operational differentiation underneath the surface similarity. TRT Diyanet Çocuk displays pronounced concentration within the conservation higher-order category (tradition at SM = 2.50) and within self-transcendence (benevolence). TRT Çocuk displays concentration within self-enhancement (achievement at SM = 1.37). The two opposing higher-order value axes identified by Schwartz (2012) thus mark the two hidden curricula with opposing lexical signatures. The empirical pattern substantiates Knafo and Schwartz (2009) prediction that value-communication profiles of media channels can be detected at the lexical level, here in the educational-media setting.

A sharper level of differentiation appears within self-regulation repertoires. Religious self-regulation is represented within TRT Diyanet at SM = 7.94, a large-excess magnitude, with Mann–Whitney U convergence at Holm-corrected *p* < 0.001. Secular self-regulation is represented within TRT Çocuk at SM = 1.56, within the modest-excess magnitude. Within the same CASEL self-management category, the lower analytic level carries substantially different operational content across the two channels.

The third layer is the interpretive scheme. CASEL-level surface similarity, read in isolation, can mask structural cultural-pedagogical differentiation beneath. The present study is best characterized as documenting comparable SEL target structure together with differentiated operational repertoire. The two channels share the basic categorical scaffolding of SEL; what fills that scaffolding (religious or secular self-regulation, conservation-oriented or self-enhancement-oriented values) diverges systematically.

This three-layered structure constitutes the principal methodological contribution of the study. When SEL content analyses within educational psychology are reduced to single-layer reporting, typically at the CASEL category level, cultural-pedagogical repertoire differences fall outside the analytic radar. Multi-layered analysis, moving from CASEL competencies through Schwartz value axes to self-regulation composition and collocation profiles, is required to close that blind spot. The finding also represents a methodological caution that fits within the SEL-grounded teacher preparation scheme advanced by Schonert-Reichl (2017).

### 5.2 Partial substitution of self-regulation repertoires

The most theoretically generative finding of the present analysis concerns the counter-complementary distribution of self-regulation strategies across the two channels. Two theses that the literature has typically treated as mutually exclusive can, under a single conceptual umbrella, be brought into productive dialogue, an umbrella that this study terms partial substitution. Saroglou (2011) supplementation reading holds that religious values overlap with universal prosocial values and add a further layer to secular strategies rather than displacing them. Its implicit counterpart is a displacement reading: where the religious repertoire achieves cultural dominance, the relative visibility of secular strategies diminishes. Both readings are empirically testable against comparable-corpus data of the kind compiled here, and the data in the present analysis tests both.

Both readings turn out to capture part of the empirical picture; neither offers a complete account on its own. On Saroglou’s side: secular self-regulation is not absent from TRT Diyanet. This conclusion rests on the descriptive and collocational evidence rather than on the non-significant inferential test. Secular self-regulation is present in TRT Diyanet at roughly 64% of the corresponding TRT Çocuk rate, and its collocate profile shows it in structured use rather than as incidental occurrence. The non-significant result for secular self-regulation (p_adj = 0.423) is reported for completeness and, given the limited power of the series-level design (§3.9), is not interpreted as evidence of equivalence. The Mercanya series openly models diaphragmatic breathing; the collocate profile of “nefes” (alıyor, veriyor, kendi, güveniyor) places a secular cognitive-behavioral repertoire on visible display within the religion-oriented channel. On the displacement side: the cultural dominance of the religious repertoire does, in relative terms, limit the visibility of the secular one. Within TRT Diyanet, secular self-regulation operates at approximately 64% of the corresponding TRT Çocuk frequency, contracted rather than eliminated.

The concept of partial substitution holds these two observations together within a single conceptual scheme. Two structural features define the concept. First, the addition of the religious repertoire does not erase the secular one. Both repertoires can co-occur, and within TRT Diyanet they do co-occur in the empirical distribution. Second, the cultural dominance of the religious repertoire relatively limits the visibility of the secular repertoire. This quantitative limitation is not categorical erasure; it constitutes a shift in dominance direction, a re-weighting rather than a replacement. McCullough and Willoughby's (2009) account of religious self-regulation processes provides theoretical traction for this reading: the religious repertoire foregrounds its own self-regulation mechanisms without excluding the secular alternatives. What shifts under partial substitution is lexical-pragmatic priority rather than presence or absence.

The implications for educational psychology bear directly on classroom practice. SEL curriculum designers should not assume a uniform self-regulation starting point across students. Children arrive with different repertoires, and those repertoires differ both in breadth and in internal weighting. A student arriving from a religiously dense media environment may walk into the classroom with internalized schemas of surrender, gratitude, and patience but with comparatively little exposure to breathing exercises, mindful counting, or planned postponement. Gay (2018) culturally responsive teaching approach and Moll et al. (1992) funds-of-knowledge perspective both call for this kind of recognition. Within Ladson-Billings (1995) culturally relevant pedagogy, recognition of the cultural-pedagogical repertoire that students bring to the classroom constitutes a precondition for the success of SEL intervention rather than an optional refinement of it.

These repertoires can be situated within the broader theoretical accounts of self-regulation. The religious and secular strategies observed here both operate as feedback-based control systems in the cybernetic sense (Carver and Scheier, 2011), draw on the socio-cognitive mechanisms of self-observation and self-reactive influence (Bandura, 1991), and recruit the executive-function processes that support the deliberate down-regulation of impulse (Hofmann et al., 2012). The prominence of the sabır (“patience”) pool in the religion-oriented channel is the lexical trace of precisely the delay-of-gratification capacity that Mischel et al. (1989) identified as a core developmental marker of self-regulation.

### 5.3 Same word, different pragmatic function

The collocation analyses presented in §4.7 (Figures 6, 7) extend the argument beyond the lexical-count level. Identical surface words carry different pragmatic functions across the two channels, and the pattern observed across the five target lemmas establishes that the divergence is systematic rather than isolated. The collocate profile of “Allah” within TRT Çocuk (anahtar, hey, kıl, bahçe) indicates secularized exclamation use; the profile within TRT Diyanet (razı, ver, çocuk, şükür) reflects direct religious rhetoric. The “arkadaş” lemma appears at high frequency in both channels but settles into distinct discourse structures: in-group interaction within Çocuk versus greeting formulae within Diyanet. The “nefes” lemma is embedded in a structured cognitive-behavioral exercise pattern within TRT Diyanet and in sparse, dispersed uses within TRT Çocuk. The “başarı” lemma occupies individual performance contexts within TRT Çocuk and teaching-based attainment contexts within TRT Diyanet. Across all five cases, the same surface lemma performs substantially different discourse work depending on the lexical environment in which it appears.

Stubbs (2001) semantic prosody captures the pattern theoretically. Word meaning is constructed through the lexical environment that surrounds it; the surface lemma represents only the visible portion of pragmatic content. Turkish renders the point particularly sharp because of its extensive inventory of secularized religious patterns such as the “Allah Allah” exclamation and the everyday idiomatic “of çok şükür,” which occur at high frequency across registers without carrying genuine religious reference. A raw report of religious-word frequency can therefore mislead substantially. KWIC inspection and collocation analysis are not optional analytic add-ons; they constitute indispensable complementary layers for any content analysis that aims to measure pragmatic content rather than mere lexical surface. The point converges with the methodological recommendations advanced by Linebarger and Walker (2005) and, in earlier corpus-linguistic work, by Baker (2006).

### 5.4 Pluralism without an equivalence claim

The surface similarity observed at the CASEL level invites, though it does not require, a reading in the direction of “the two content streams are comparable in SEL terms.” Such a reading occupies a legitimate empirical position within the existing literature. Saroglou and Cohen (2011) document overlap between religious values and universal prosocial values; King and Boyatzis (2015) press for the integration of religious development with mainstream developmental psychology. The surface similarity is, in that sense, not a false signal. The drift toward a naive equivalence claim, however, must be avoided, and the analytic structure of the present study is designed precisely to prevent that drift. Three principles articulate where the interpretive boundary properly sits.

#### 5.4.1 Principle 1: comparable target structure differs from equivalent operational content

The surface similarity observed at the CASEL level does not entail that the two content streams deliver the same content in different packages. The two streams diverge systematically along Schwartz value axes and self-regulation repertoires. Within the same CASEL self-management category, one channel reaches for “calm down,” “breathe,” and “plan”; the other reaches for “be patient,” “give thanks,” “surrender,” and “pray.” These contents are not interchangeable substitutes for one another. Each carries its own epistemic and pragmatic frame.

#### 5.4.2 Principle 2: the partial substitution finding provides structural insurance against the equivalence claim

The partial substitution finding reported in §4.3 and §5.2 empirically refutes any naive equivalence claim. Religious self-regulation appears within TRT Diyanet at SM = 7.94 (large excess); secular self-regulation appears within TRT Çocuk at SM = 1.56 (modest excess). The two channels do not deliver the same amount of self-regulation in different packages; the repertoires diverge in their internal composition. This constitutes the careful version of a pluralistic reading: different cultural-pedagogical repertoires contribute to shared SEL targets through different operational pathways, but those pathways are neither reducible to one another nor fully substitutable.

#### 5.4.3 Principle 3: the study carries no normative claim

The findings remain descriptive and analytic throughout. No evaluative judgment is offered between the two content streams, and no recommendation is made to policy-makers or to parents in favor of one content type over the other. The practical proposals set out in §5.5 are culturally responsive teaching suggestions built on the empirical reality that both content types already exist within the children’s media environment.

### 5.5 Practical implications

The findings bear on four practical areas of educational psychology.

At the level of SEL curriculum design, classroom SEL programs should recognize the two distinct self-regulation repertoires that students may bring with them. The semantic links between them should be explicitly bridged: the parallel between patience and postponement; the link between gratitude and mindful awareness; the overlap between surrender and acceptance-based cognitive techniques. This bridging is required so that students from religion-oriented backgrounds can access secular techniques without alienation, and so that students from secular backgrounds can understand the conceptual structure of the religious repertoire.

At the level of teacher preparation, Schonert-Reichl (2017) SEL-grounded teacher preparation scheme should include awareness of the informal self-regulation baggage students may bring as an addable component. Teacher candidates should be aware that children from religiously dense media or family environments arrive in the classroom with particular lexical-pragmatic schemas, and that those schemas do not map one-to-one onto secular SEL terms. The operationalization of Ladson-Billings (1995) culturally relevant pedagogy requires this awareness.

At the level of parent and educator content-selection guidance, the findings do not put forward an evaluative judgment between the two channels. They map transparently which lexical-semantic repertoire each channel offers at what density. Parents and educators can make choices according to their own pedagogical preferences; the study supplies empirical information for that choice and does not give normative direction.

At the level of integrating religious-content analysis into educational psychology, the integration proposed by King and Boyatzis (2015) and Saroglou (2011) can be carried out by comparative corpus methods, as the present study has substantiated. The approach offers one answer to the issue of methodological standardization of religious content analysis that Pargament et al. (2013) flagged in the APA Handbook of Psychology, Religion, and Spirituality.

### 5.6 Methodological assessment and sample-size limits

The study was carried out using standard corpus-linguistic measures (with Sketch Engine, Wordsmith Tools) over morphologically lemmatized Turkish text. Standard measures (fpmw, Simple Maths, G^2^, logDice) combined with Gries’s DP dispersion check and systematic KWIC inspection allow the findings to be read transparently in both magnitude and context. Excluding character and program names from the keyness filter is a standard noise-cleaning step in content analysis (Baker, 2006; Brezina, 2018) and is documented in Appendix A.

The construct-validity standard applied throughout the analysis is that of corpus-based content analysis rather than that of individual-differences psychometrics. The indicator pools measure lexical-density signatures of theory-grounded constructs within broadcast content; the pools are anchored to Turkish-validated psychometric scales (Totan, 2018; Demirutku and Sümer, 2010) but operationalized through morphological expansion and contextual KWIC verification rather than through scale-item administration to participants. This approach is appropriate for input-level characterization and consistent with established practice in corpus-based content analysis. The approach should not, however, be conflated with person-level measurement of CASEL competencies or Schwartz values. Reception-level studies (§5.7) would be required to connect input-level lexical density to person-level construct realization in viewers.

Sample size constitutes the most visible limit on the present analysis. The two-channel × 12-series design yields *n* = 12 for Mann–Whitney *U* tests at the series level. For medium-to-large effect sizes the sample provides acceptable statistical power; religious vocabulary (|*r*| = 0.82) and religious self-regulation (|*r*| = 0.96) raise no power-related concerns at these large magnitudes. For small-to-medium effects, however, the sample is thin. The null result on benevolence cannot be distinguished, on the present sample, between a genuine null effect and an inadequate-power outcome; that question remains formally open and would benefit from replication with a larger series sample. The sample is also restricted to TRT’s two channels; private-sector children’s channels (Disney Çocuk, Cartoon Network Turkey, Minika Çocuk) fall outside the design. The generalizability of the findings holds for the “public broadcasting dual-channel configuration” rather than for “Turkish children’s media” as a whole.

Several further structural limits warrant mention. Content analysis does not measure reception: how children interpret, internalize, or push back against the content lies outside the scope of the present analysis. No causal claim is offered or implied. Lemmatization can introduce small variance across morphological processing tools; the systematic KWIC inspection layer balances that sensitivity without eliminating it entirely. The absence of a sufficiently strong Turkish dependency parser ruled out grammatical-relation-based word sketch analysis, and ±5 window collocation analysis was substituted in its place. That methodological adjustment is documented in §3.5 and Appendix C and is presented here as a limitation rather than as a methodological preference.

### 5.7 From input to reception: a research program

The present analysis sits at the content-characterization level and carries no reception claim. The limit reflects an epistemic position rather than a methodological preference; content characterization functions as the necessary first step toward reception research, not as a workable substitute for it. The historical arc of Mares and Pan's (2013) meta-analysis of Sesame Street demonstrates the temporal logic of this position: the first Sesame Street content analyses were carried out during the 1970s, while systematic reception meta-analysis only became feasible during the 2010s. The present study is positioned as the first step of a parallel research program within the TRT dual-channel configuration.

Three designs could carry this program forward. The first design is correlational: the relation between measured channel-usage frequency and adoption of self-regulation strategies can be tested through Demirutku and Sümer (2010) Turkish value scale and Totan (2018) Turkish SEL scale. The second design is short-term interventional: presentation of the same content to children in controlled settings with immediate measurement of value-flow internalization. The third design is longitudinal: tracking the effects of parental-usage profiles on child SEL development at yearly intervals. The three designs taken together can connect the present descriptive input characterization to a causal research line.

A second extension concerns the evaluative layer of the same material. The present analysis characterizes *what* is said by measuring the lexical density of theory-derived constructs; it does not code how those constructs are evaluatively positioned. Martin and White (2005) Appraisal framework offers the appropriate instrument for that step. Its distinction among attitude, engagement, and graduation would make visible whether *sabır* (“patience”) or *nefes* (“breath”) is presented as obligation, as achievement, or as affect — a distinction that frequency and collocation evidence can indicate but not settle. Applying the framework here would require attitude-level annotation of the full transcript set, a coding layer beyond the scope of the present study. An Appraisal-based re-reading of the two sub-corpora is therefore identified as a natural companion to the reception program outlined above.

### 5.8 Directions for future research

Beyond the reception designs above, three additional research directions can be considered. The first is comparative extension: similar comparable-corpus designs in other cultural-religious settings (Indonesia, Iran, Pakistan, Israel) can test for cross-cultural occurrence of the partial substitution pattern. The second is grammatical-relation analysis: once a stronger Turkish dependency parser becomes available, Word Sketch-based grammatical collocation analysis can be applied. The third is cross-media extension: children’s books, digital games, and social media content can be examined with the same analytic pipeline.

## 6 Conclusion

A comparison of 225,451 matched tokens from two Turkish public children’s broadcasters, run with standard corpus-linguistic metrics, documents two different cultural operationalizations of SEL as hidden curriculum. The basic category structure of the CASEL competencies is broadly distributed in both channels; if read on its own, that surface similarity can drift toward a naive equivalence claim. The Schwartz value axes, self-regulation repertoires, and collocation profiles bring out a structural cultural-pedagogical differentiation underneath the surface similarity. This three-layered finding substantiates the need for a multi-layered approach in SEL content analysis within educational psychology and shows that the informal-curriculum profiles children bring can be quantitatively described at the lexical-semantic level.

The study contributes empirically to the input-characterization line of educational psychology in the Turkish educational-media setting on four levels: methodological (an open, replicable operationalization of standard corpus-linguistic measuresand softwares); theoretical (the partial substitution concept); empirical (the first systematic comparison of two broadcasting policies); and practical (an empirical foundation for culturally responsive SEL curriculum design). The findings show that religious and secular SEL operationalizations are not mutually exclusive but differ in composition as cultural-pedagogical repertoires. This view can serve as an empirical building block in integrating the religious development and culturally responsive pedagogy sub-lines of educational psychology into the mainstream.

## Data Availability

The original contributions presented in the study are included in the article/Supplementary material, further inquiries can be directed to the corresponding author.
