# Supplementary material for: Partial substitution in the hidden curriculum of children’s media: a comparative corpus analysis of religion-oriented and general-educational Turkish public broadcasting
Source: Front Psychol. 2026 Aug 25;17:1887219. doi: 10.3389/fpsyg.2026.1887219 (PMC13549891; doi:10.3389/fpsyg.2026.1887219)
Supplement: Supplementary file 1 [file supplementary_file_1.docx]

**Supplementary Material**

*Appendix A. Indicator-Pool Lemma Frequencies (Corrected Morphological Analysis)*

This appendix reports the frequency distribution of the indicator pools under the corrected analysis. Pool membership was determined at the lemma level with a rule-based morphological analyzer for Turkish, using the study's theory-derived seed terms, with privative and negation-suffixed forms excluded. For each pool the following are reported: frequency per million words (fpmw) by channel, Simple Maths keyness (SM), the log-likelihood statistic G², and Gries's DP. Component lemma frequencies are raw counts and may overlap where a token satisfies more than one seed; pool-level statistics are computed over the deduplicated pool.

**C1. CASEL: Self-Awareness**

*TRT Çocuk = 10,408.6 fpmw; TRT Diyanet Çocuk = 9,905.0 fpmw; SM = 1.05; G² = 1.4; DP = 0.128.*

| **Lemma** | **TRT Çocuk** | **TRT Diyanet Çocuk** | **Total** |
| --- | --- | --- | --- |
| anla | 610 | 548 | 1,158 |
| kendi | 220 | 206 | 426 |
| farkına | 91 | 55 | 146 |
| güçlü | 31 | 99 | 130 |
| düşün | 52 | 51 | 103 |
| hayal | 62 | 38 | 100 |
| değer | 44 | 32 | 76 |
| duygu | 7 | 46 | 53 |
| hisset | 16 | 22 | 38 |
| amaç | 12 | 5 | 17 |
| umut | 14 | 3 | 17 |
| düşünce | 9 | 2 | 11 |
| hedef | 6 | 3 | 9 |
| zayıf | 1 | 5 | 6 |
| inanç | 1 | 0 | 1 |

**C2. CASEL: Self-Management**

*TRT Çocuk = 2,690.7 fpmw; TRT Diyanet Çocuk = 2,125.0 fpmw; SM = 1.27; G² = 7.5; DP = 0.160.*

| **Lemma** | **TRT Çocuk** | **TRT Diyanet Çocuk** | **Total** |
| --- | --- | --- | --- |
| sakin | 65 | 55 | 120 |
| kararlı | 43 | 34 | 77 |
| kontrol | 58 | 14 | 72 |
| plan | 50 | 9 | 59 |
| dayan | 24 | 25 | 49 |
| vazgeç | 24 | 12 | 36 |
| sabır | 12 | 22 | 34 |
| düzen | 8 | 25 | 33 |
| öfke | 2 | 15 | 17 |
| stres | 1 | 16 | 17 |
| motivasyon | 13 | 2 | 15 |
| sabret | 2 | 8 | 10 |
| azim | 1 | 2 | 3 |
| disiplin | 1 | 0 | 1 |

**C3. CASEL: Social Awareness**

*TRT Çocuk = 6,337.2 fpmw; TRT Diyanet Çocuk = 7,015.3 fpmw; SM = 1.11; G² = 3.9; DP = 0.103.*

| **Lemma** | **TRT Çocuk** | **TRT Diyanet Çocuk** | **Total** |
| --- | --- | --- | --- |
| anlamak | 358 | 358 | 716 |
| yardım | 194 | 268 | 462 |
| farklı | 91 | 55 | 146 |
| kabul | 28 | 37 | 65 |
| acım | 17 | 20 | 37 |
| çeşit | 18 | 19 | 37 |
| saygı | 5 | 10 | 15 |
| merhamet | 0 | 14 | 14 |
| anlayış | 4 | 3 | 7 |
| yardımsever | 1 | 4 | 5 |
| şefkat | 0 | 1 | 1 |

**C4. CASEL: Relationship Skills**

*TRT Çocuk = 13,152.4 fpmw; TRT Diyanet Çocuk = 11,878.9 fpmw; SM = 1.11; G² = 7.3; DP = 0.114.*

| **Lemma** | **TRT Çocuk** | **TRT Diyanet Çocuk** | **Total** |
| --- | --- | --- | --- |
| arkadaş | 312 | 219 | 531 |
| yardımlaş | 194 | 268 | 462 |
| anlaş | 252 | 190 | 442 |
| dost | 140 | 166 | 306 |
| dinle | 130 | 91 | 221 |
| uzlaş | 151 | 48 | 199 |
| konuş | 77 | 116 | 193 |
| birlikte | 84 | 89 | 173 |
| beraber | 53 | 23 | 76 |
| paylaş | 28 | 36 | 64 |
| ekip | 13 | 31 | 44 |
| çözüm | 20 | 14 | 34 |
| destek | 9 | 21 | 30 |
| barış | 11 | 14 | 25 |
| kucakla | 3 | 8 | 11 |
| iletişim | 9 | 1 | 10 |
| işbirliği | 0 | 1 | 1 |

**C5. CASEL: Responsible Decision-Making**

*TRT Çocuk = 11,665.5 fpmw; TRT Diyanet Çocuk = 15,311.0 fpmw; SM = 1.31; G² = 55.7; DP = 0.161.*

| **Lemma** | **TRT Çocuk** | **TRT Diyanet Çocuk** | **Total** |
| --- | --- | --- | --- |
| iyi | 659 | 913 | 1,572 |
| doğru | 216 | 254 | 470 |
| hak | 116 | 320 | 436 |
| yanlış | 75 | 61 | 136 |
| kötü | 71 | 36 | 107 |
| düşün | 52 | 51 | 103 |
| karar | 43 | 34 | 77 |
| sonuç | 49 | 19 | 68 |
| sorumlu | 14 | 14 | 28 |
| etki | 20 | 6 | 26 |
| sorumluluk | 11 | 14 | 25 |
| ahlak | 0 | 7 | 7 |
| vicdan | 0 | 3 | 3 |
| adil | 3 | 0 | 3 |
| adalet | 0 | 2 | 2 |
| tart | 0 | 2 | 2 |

**S1. Schwartz: Self-Direction**

*TRT Çocuk = 5,859.3 fpmw; TRT Diyanet Çocuk = 5,157.0 fpmw; SM = 1.14; G² = 5.0; DP = 0.124.*

| **Lemma** | **TRT Çocuk** | **TRT Diyanet Çocuk** | **Total** |
| --- | --- | --- | --- |
| kendin | 220 | 206 | 426 |
| merak | 187 | 117 | 304 |
| fikir | 86 | 127 | 213 |
| öğren | 83 | 65 | 148 |
| yarat | 54 | 48 | 102 |
| yaratıcı | 54 | 48 | 102 |
| keşfet | 17 | 4 | 21 |
| keşif | 10 | 11 | 21 |
| özgür | 4 | 2 | 6 |
| özgürlük | 1 | 0 | 1 |
| bağımsızlık | 1 | 0 | 1 |

**S2. Schwartz: Stimulation**

*TRT Çocuk = 3,407.6 fpmw; TRT Diyanet Çocuk = 2,765.2 fpmw; SM = 1.23; G² = 7.5; DP = 0.201.*

| **Lemma** | **TRT Çocuk** | **TRT Diyanet Çocuk** | **Total** |
| --- | --- | --- | --- |
| yeni | 244 | 144 | 388 |
| heyecan | 60 | 73 | 133 |
| macera | 42 | 80 | 122 |
| değişik | 17 | 7 | 24 |
| cesur | 9 | 5 | 14 |
| cesaret | 9 | 2 | 11 |
| risk | 4 | 0 | 4 |
| yenilik | 1 | 0 | 1 |

**S3. Schwartz: Hedonism**

*TRT Çocuk = 3,354.5 fpmw; TRT Diyanet Çocuk = 3,565.5 fpmw; SM = 1.06; G² = 0.7; DP = 0.227.*

| **Lemma** | **TRT Çocuk** | **TRT Diyanet Çocuk** | **Total** |
| --- | --- | --- | --- |
| hoş | 85 | 95 | 180 |
| tat | 73 | 88 | 161 |
| eğlence | 84 | 37 | 121 |
| tatlı | 48 | 65 | 113 |
| mutluluk | 53 | 57 | 110 |
| keyif | 25 | 53 | 78 |
| eğlen | 33 | 36 | 69 |
| lezzet | 15 | 21 | 36 |
| zevk | 10 | 7 | 17 |
| haz | 1 | 7 | 8 |

**S4. Schwartz: Achievement**

*TRT Çocuk = 2,973.9 fpmw; TRT Diyanet Çocuk = 2,178.4 fpmw; SM = 1.37; G² = 13.9; DP = 0.177.*

| **Lemma** | **TRT Çocuk** | **TRT Diyanet Çocuk** | **Total** |
| --- | --- | --- | --- |
| akıllı | 85 | 121 | 206 |
| üstün | 43 | 46 | 89 |
| kazan | 44 | 17 | 61 |
| başar | 45 | 15 | 60 |
| ödül | 41 | 10 | 51 |
| yetenek | 24 | 26 | 50 |
| başarı | 23 | 10 | 33 |
| rekor | 24 | 2 | 26 |
| zafer | 15 | 1 | 16 |

**S5. Schwartz: Power**

*TRT Çocuk = 1,071.0 fpmw; TRT Diyanet Çocuk = 1,458.2 fpmw; SM = 1.36; G² = 6.7; DP = 0.428.*

| **Lemma** | **TRT Çocuk** | **TRT Diyanet Çocuk** | **Total** |
| --- | --- | --- | --- |
| güç | 107 | 127 | 234 |
| güçlü | 31 | 99 | 130 |
| zengin | 6 | 29 | 35 |
| lider | 3 | 4 | 7 |
| servet | 0 | 3 | 3 |
| hâkim | 2 | 0 | 2 |
| yöneti | 1 | 1 | 2 |
| yönetici | 2 | 0 | 2 |

**S6. Schwartz: Security**

*TRT Çocuk = 2,885.4 fpmw; TRT Diyanet Çocuk = 4,143.4 fpmw; SM = 1.44; G² = 25.5; DP = 0.230.*

| **Lemma** | **TRT Çocuk** | **TRT Diyanet Çocuk** | **Total** |
| --- | --- | --- | --- |
| emin | 95 | 202 | 297 |
| sağlıklı | 120 | 142 | 262 |
| sağlık | 39 | 51 | 90 |
| kararlı | 43 | 34 | 77 |
| güven | 35 | 23 | 58 |
| koruma | 19 | 30 | 49 |
| huzur | 8 | 21 | 29 |
| koru | 3 | 15 | 18 |
| düzenli | 1 | 13 | 14 |
| güvenlik | 9 | 1 | 10 |
| emniyet | 2 | 1 | 3 |
| savun | 2 | 0 | 2 |
| savunma | 1 | 0 | 1 |
| istikrar | 1 | 0 | 1 |

**S7. Schwartz: Conformity**

*TRT Çocuk = 265.5 fpmw; TRT Diyanet Çocuk = 302.3 fpmw; SM = 1.14; G² = 0.3; DP = 0.405.*

| **Lemma** | **TRT Çocuk** | **TRT Diyanet Çocuk** | **Total** |
| --- | --- | --- | --- |
| kural | 22 | 13 | 35 |
| saygı | 5 | 10 | 15 |
| uyum | 1 | 9 | 10 |
| terbiye | 1 | 2 | 3 |
| itaat | 1 | 0 | 1 |

**S8. Schwartz: Tradition**

*TRT Çocuk = 3,354.5 fpmw; TRT Diyanet Çocuk = 8,375.7 fpmw; SM = 2.50; G² = 250.4; DP = 0.248.*

| **Lemma** | **TRT Çocuk** | **TRT Diyanet Çocuk** | **Total** |
| --- | --- | --- | --- |
| allah | 81 | 552 | 633 |
| atalar | 130 | 88 | 218 |
| din | 87 | 64 | 151 |
| dini | 87 | 64 | 151 |
| tarih | 23 | 21 | 44 |
| tarihi | 23 | 21 | 44 |
| namaz | 0 | 44 | 44 |
| peygamber | 1 | 36 | 37 |
| gelenek | 10 | 26 | 36 |
| ata | 16 | 13 | 29 |
| millet | 9 | 13 | 22 |
| milli | 17 | 2 | 19 |
| hac | 1 | 18 | 19 |
| cennet | 3 | 12 | 15 |
| adet | 6 | 7 | 13 |
| isa | 0 | 11 | 11 |
| ayet | 1 | 7 | 8 |
| kültür | 3 | 3 | 6 |
| hazreti | 0 | 6 | 6 |
| sünnet | 0 | 5 | 5 |
| musa | 0 | 5 | 5 |
| töre | 1 | 3 | 4 |
| iman | 1 | 3 | 4 |
| ibadet | 0 | 4 | 4 |
| rab | 0 | 3 | 3 |
| ibrahim | 0 | 3 | 3 |
| hadis | 0 | 2 | 2 |
| kuran | 1 | 1 | 2 |
| miras | 1 | 0 | 1 |
| köken | 1 | 0 | 1 |
| inanç | 1 | 0 | 1 |
| zekat | 0 | 1 | 1 |
| rahim | 1 | 0 | 1 |
| muhammed | 0 | 1 | 1 |
| kıyamet | 0 | 1 | 1 |

**S9. Schwartz: Benevolence**

*TRT Çocuk = 8,585.4 fpmw; TRT Diyanet Çocuk = 11,630.0 fpmw; SM = 1.35; G² = 51.9; DP = 0.165.*

| **Lemma** | **TRT Çocuk** | **TRT Diyanet Çocuk** | **Total** |
| --- | --- | --- | --- |
| iyi | 659 | 913 | 1,572 |
| yardım | 194 | 268 | 462 |
| sevgi | 64 | 45 | 109 |
| sevgili | 55 | 27 | 82 |
| affet | 20 | 30 | 50 |
| dostluk | 27 | 9 | 36 |
| bağışla | 2 | 14 | 16 |
| merhamet | 0 | 14 | 14 |
| sadık | 2 | 9 | 11 |
| vefa | 1 | 4 | 5 |
| şefkat | 0 | 1 | 1 |
| sadakat | 0 | 1 | 1 |
| fedakâr | 1 | 0 | 1 |

**S10. Schwartz: Universalism**

*TRT Çocuk = 2,965.0 fpmw; TRT Diyanet Çocuk = 3,103.1 fpmw; SM = 1.05; G² = 0.4; DP = 0.294.*

| **Lemma** | **TRT Çocuk** | **TRT Diyanet Çocuk** | **Total** |
| --- | --- | --- | --- |
| insanlık | 119 | 158 | 277 |
| dünya | 118 | 98 | 216 |
| doğa | 40 | 34 | 74 |
| doğal | 13 | 33 | 46 |
| çevre | 24 | 6 | 30 |
| barış | 11 | 14 | 25 |
| eşit | 4 | 3 | 7 |
| adil | 3 | 0 | 3 |
| hoşgörü | 3 | 0 | 3 |
| adalet | 0 | 2 | 2 |
| evren | 0 | 1 | 1 |

**RV. Theme: Religious / Spiritual Vocabulary**

*TRT Çocuk = 4,372.3 fpmw; TRT Diyanet Çocuk = 14,030.7 fpmw; SM = 3.21; G² = 601.3; DP = 0.362.*

| **Lemma** | **TRT Çocuk** | **TRT Diyanet Çocuk** | **Total** |
| --- | --- | --- | --- |
| allah | 81 | 552 | 633 |
| hoca | 139 | 146 | 285 |
| din | 87 | 64 | 151 |
| dini | 87 | 64 | 151 |
| inşallah | 6 | 104 | 110 |
| şükür | 5 | 84 | 89 |
| cami | 8 | 72 | 80 |
| selam | 22 | 50 | 72 |
| bayram | 59 | 1 | 60 |
| maşallah | 3 | 56 | 59 |
| yakar | 34 | 20 | 54 |
| helal | 5 | 44 | 49 |
| namaz | 0 | 44 | 44 |
| peygamber | 1 | 36 | 37 |
| dua | 5 | 25 | 30 |
| kandil | 2 | 26 | 28 |
| bismillah | 0 | 25 | 25 |
| minare | 1 | 23 | 24 |
| tövbe | 1 | 20 | 21 |
| hac | 1 | 18 | 19 |
| elhamdülillah | 0 | 18 | 18 |
| hafız | 12 | 6 | 18 |
| ezan | 0 | 17 | 17 |
| cennet | 3 | 12 | 15 |
| abdest | 0 | 14 | 14 |
| bereket | 2 | 10 | 12 |
| isa | 0 | 11 | 11 |
| ramazan | 9 | 2 | 11 |
| imam | 0 | 10 | 10 |
| ayet | 1 | 7 | 8 |
| sevap | 0 | 7 | 7 |
| hazreti | 0 | 6 | 6 |
| musa | 0 | 5 | 5 |
| sünnet | 0 | 5 | 5 |
| günah | 3 | 2 | 5 |
| şükret | 0 | 5 | 5 |
| ibadet | 0 | 4 | 4 |
| kıble | 0 | 4 | 4 |
| iman | 1 | 3 | 4 |
| rab | 0 | 3 | 3 |
| ibrahim | 0 | 3 | 3 |
| müezzin | 0 | 3 | 3 |
| hadis | 0 | 2 | 2 |
| kuran | 1 | 1 | 2 |
| sure | 0 | 2 | 2 |
| mescit | 0 | 2 | 2 |
| rahim | 1 | 0 | 1 |
| muhammed | 0 | 1 | 1 |
| zekat | 0 | 1 | 1 |
| kıyamet | 0 | 1 | 1 |
| inanç | 1 | 0 | 1 |
| tevekkül | 0 | 1 | 1 |

**R1. Theme: Religious Self-Regulation**

*TRT Çocuk = 947.0 fpmw; TRT Diyanet Çocuk = 7,531.0 fpmw; SM = 7.94; G² = 656.2; DP = 0.439.*

| **Lemma** | **TRT Çocuk** | **TRT Diyanet Çocuk** | **Total** |
| --- | --- | --- | --- |
| allaha | 81 | 552 | 633 |
| razı | 1 | 95 | 96 |
| şükür | 5 | 84 | 89 |
| sabır | 12 | 22 | 34 |
| dua | 5 | 25 | 30 |
| tövbe | 1 | 20 | 21 |
| rabbim | 0 | 18 | 18 |
| rıza | 0 | 17 | 17 |
| sabret | 2 | 8 | 10 |
| şükret | 0 | 5 | 5 |
| tevekkül | 0 | 1 | 1 |

**R2. Theme: Secular Self-Regulation**

*TRT Çocuk = 2,717.2 fpmw; TRT Diyanet Çocuk = 1,742.7 fpmw; SM = 1.56; G² = 24.2; DP = 0.187.*

| **Lemma** | **TRT Çocuk** | **TRT Diyanet Çocuk** | **Total** |
| --- | --- | --- | --- |
| sakin | 65 | 55 | 120 |
| kararlı | 43 | 34 | 77 |
| kontrol | 58 | 14 | 72 |
| plan | 50 | 9 | 59 |
| say | 24 | 26 | 50 |
| vazgeçme | 24 | 12 | 36 |
| nefes | 13 | 20 | 33 |
| öfkemi | 2 | 15 | 17 |
| motivasyon | 13 | 2 | 15 |
| odaklan | 10 | 4 | 14 |
| konsantre | 2 | 3 | 5 |
| azim | 1 | 2 | 3 |
| disiplin | 1 | 0 | 1 |
| motive | 1 | 0 | 1 |

**T1. Theme: Trust Emotion**

*TRT Çocuk = 1,832.1 fpmw; TRT Diyanet Çocuk = 2,658.5 fpmw; SM = 1.45; G² = 17.2; DP = 0.363.*

| **Lemma** | **TRT Çocuk** | **TRT Diyanet Çocuk** | **Total** |
| --- | --- | --- | --- |
| emin | 95 | 202 | 297 |
| rahat | 49 | 52 | 101 |
| güven | 35 | 23 | 58 |
| inan | 12 | 17 | 29 |
| umut | 14 | 3 | 17 |
| emniyet | 2 | 1 | 3 |
| güvenilir | 0 | 1 | 1 |

**E1. Theme: General Emotion**

*TRT Çocuk = 7,868.4 fpmw; TRT Diyanet Çocuk = 7,682.2 fpmw; SM = 1.02; G² = 0.3; DP = 0.133.*

| **Lemma** | **TRT Çocuk** | **TRT Diyanet Çocuk** | **Total** |
| --- | --- | --- | --- |
| merak | 187 | 117 | 304 |
| sevin | 131 | 111 | 242 |
| kız | 167 | 59 | 226 |
| kork | 49 | 84 | 133 |
| mutluluk | 53 | 57 | 110 |
| mutlu | 48 | 56 | 104 |
| rahat | 49 | 52 | 101 |
| üzül | 38 | 62 | 100 |
| keyif | 25 | 53 | 78 |
| şaşır | 27 | 30 | 57 |
| duygu | 7 | 46 | 53 |
| üzgün | 24 | 17 | 41 |
| hisset | 16 | 22 | 38 |
| neşe | 22 | 14 | 36 |
| korku | 12 | 20 | 32 |
| endişe | 21 | 10 | 31 |
| huzur | 8 | 21 | 29 |
| hayret | 8 | 13 | 21 |
| gurur | 10 | 9 | 19 |
| öfke | 2 | 15 | 17 |
| sinir | 12 | 4 | 16 |
| utan | 1 | 12 | 13 |
| mahcup | 2 | 10 | 12 |
| şaşkın | 4 | 6 | 10 |
| pişman | 5 | 5 | 10 |
| kızgın | 3 | 5 | 8 |
| sevinç | 1 | 6 | 7 |
| his | 2 | 3 | 5 |
| üzüntü | 0 | 2 | 2 |
| kaygı | 2 | 0 | 2 |
| tasa | 1 | 0 | 1 |
