# Supplementary material for: Partial substitution in the hidden curriculum of children’s media: a comparative corpus analysis of religion-oriented and general-educational Turkish public broadcasting
Source: Front Psychol. 2026 Aug 25;17:1887219. doi: 10.3389/fpsyg.2026.1887219 (PMC13549891; doi:10.3389/fpsyg.2026.1887219)
Supplement: Supplementary file 2 [file supplementary_file_2.docx]

**Appendix B. Series-Level Token Counts**

This appendix reports the token count of each series in the two sub-corpora, tokenized with corpus softare (Word Smithtools, Sketch Engine). Series are listed in descending order of size. The channel totals match the corpus sizes given in Section 3.3 and in Table 1 (TRT Çocuk = 112,983 tokens across 12 series; TRT Diyanet Çocuk = 112,468 tokens across 12 series; 225,451 tokens in total).

**TRT Çocuk**

| **Series** | **Tokens** |
| --- | --- |
| Rafadan Tayfa | 20,887 |
| Keloğlan | 13,777 |
| İbi | 9,988 |
| Dijital Tayfa | 9,607 |
| Kehribar Boncuk | 8,946 |
| Z Takımı | 8,819 |
| Aslan | 8,339 |
| Nasreddin Hoca Zaman Yolcusu | 7,991 |
| Elif | 7,958 |
| Ege ile Gaga | 5,876 |
| Maysa | 5,656 |
| Niloya | 5,139 |
| **Total** | **112,983** |

**TRT Diyanet Çocuk**

| **Series** | **Tokens** |
| --- | --- |
| Küçük Hafız Emin | 16,867 |
| Hay Hak | 14,104 |
| İyilik Habercileri | 11,332 |
| Heybeli Tiyatro | 10,988 |
| Mercanya | 9,501 |
| Fül Fül Hac Yolunda | 9,236 |
| Gezgin Çelebi | 8,254 |
| Çufi | 7,551 |
| Miş Miş | 7,548 |
| Cümbür Cemaat | 7,532 |
| Zekice İşler | 6,228 |
| Ayrı Telden | 3,327 |
| **Total** | **112,468** |

*Note. Counts are word tokens under the study's morphological tokenization; forms are resolved to dictionary lemmas before counting. Series-level values sum exactly to the channel totals reported in Section 3.3 and Table 1. Series titles follow the on-screen broadcast names.*
