# Supplementary material for: Partial substitution in the hidden curriculum of children’s media: a comparative corpus analysis of religion-oriented and general-educational Turkish public broadcasting
Source: Front Psychol. 2026 Aug 25;17:1887219. doi: 10.3389/fpsyg.2026.1887219 (PMC13549891; doi:10.3389/fpsyg.2026.1887219)
Supplement: Supplementary file 3 [file supplementary_file_3.docx]

**Appendix C. Interpretation Guides for the Corpus Measures**

This appendix specifies how each corpus-linguistic measure used in the study is computed and how its values are interpreted. It is intended as a reading key for the statistics reported in Sections 3.5 to 4.7 and in Appendices A and D. All measures are computed over the two balanced sub-corpora after tokenization and lemmatization for Turkish described in Section 3.5; forms carrying privative or negation suffixes are excluded before counting.

**C.1 Simple Maths keyness (SM)**

Simple Maths quantifies how much more frequent a category is in one channel than in the other, using the normalized (per-million-word) frequencies with a smoothing parameter N = 1 (Kilgarriff, 2009). A focus and a reference channel are specified, and the ratio is read against the interpretive bands of Brezina (2018):

| **SM value** | **Interpretation** |
| --- | --- |
| ≤ 2 | modest excess |
| > 2 and ≤ 5 | moderate-to-pronounced excess |
| > 5 | large excess |

Keyness is directional: the channel named as focus is the one in which the category is enriched. The direction is reported alongside each SM value in the main text and in Appendix A.

**C.2 Log-likelihood (G²)**

The log-likelihood statistic G² (Dunning, 1993) is reported alongside each keyness contrast as a corroborating significance indicator for the size of the frequency difference. Larger G² values indicate that the observed difference is less likely under the null of equal proportions. G² is used descriptively, to confirm the direction and magnitude given by Simple Maths, rather than as the primary inferential test; the pre-registered inferential tests are reported in Appendix D.

**C.3 Collocation: logDice and mutual information**

Collocation is analyzed within a ±5-token window around each target lemma. Two association measures are reported. logDice (Rychlý, 2008) is a scale-independent measure whose values do not depend on corpus size, which makes it suitable for comparing collocational strength across the two channels; higher values indicate a stronger association. Mutual information (Church & Hanks, 1990) is reported as a complementary measure that highlights exclusive pairings but is more sensitive to low-frequency items. Collocates are inspected in the concordance before interpretation.

**C.4 Dispersion: Gries's deviation of proportions (DP)**

Dispersion across the series within a channel is measured with Gries's deviation of proportions (Gries, 2008). DP ranges from 0 to 1 and indicates how evenly a category is spread across the series of a channel:

| **DP value** | **Interpretation** |
| --- | --- |
| < 0.5 | channel-distributed (spread across series) |
| ≥ 0.5 | series-specific (concentrated in few series) |

A low DP indicates that a category reflects a channel-level pattern rather than the idiosyncrasy of one or two series. Where a category shows a high raw frequency but a DP approaching the 0.5 threshold, the main text flags the value explicitly and interprets it with corresponding caution.
