# Supplementary material for: Partial substitution in the hidden curriculum of children’s media: a comparative corpus analysis of religion-oriented and general-educational Turkish public broadcasting
Source: Front Psychol. 2026 Aug 25;17:1887219. doi: 10.3389/fpsyg.2026.1887219 (PMC13549891; doi:10.3389/fpsyg.2026.1887219)
Supplement: Supplementary file 4 [file supplementary_file_4.docx]

**Appendix D. Full Inferential Results**

This appendix reports the complete inferential test results for the five pre-registered hypotheses. Each contrast compares the two channels on the series-level distribution of the relevant lemma pool (n = 12 series per channel), using the Mann–Whitney U test with the pre-registered directionality. Reported p-values are adjusted for multiple comparisons with the Bonferroni–Holm procedure across the family of five tests. Effect sizes are the rank-biserial correlation |r|. Decisions are taken at α = .05 on the Holm-adjusted p-value.

| **H** | **Contrast (lemma pool)** | **Test** | **U** | **p (raw)** | **p (Holm)** | **\|r\|** |
| --- | --- | --- | --- | --- | --- | --- |
| H1 | Religious / spiritual vocabulary | one-tailed (greater) | 131 | <.001 | .001 | .82 |
| H2 | Religious self-regulation | one-tailed (greater) | 141 | <.001 | <.001 | .96 |
| H3 | Schwartz benevolence | one-tailed (greater) | 85 | .235 | .470 | .18 |
| H4 | Trust emotion | one-tailed (greater) | 69 | .580 | .580 | .04 |
| H5 | Secular self-regulation | two-tailed | 46 | .141 | .423 | .36 |

Decisions (Holm-adjusted, α = .05): H1 reject H₀; H2 reject H₀; H3 retain H₀; H4 retain H₀; H5 retain H₀. The two rejections (religious vocabulary and religious self-regulation) are the large-magnitude contrasts on which the substantive claims rest; the three retained nulls (benevolence, trust emotion, secular self-regulation) are interpreted descriptively, not as evidence of equivalence.

**Statistical power**

Because the unit of analysis is the series (n = 12 per channel), the design detects only large between-channel effects. For a two-tailed Mann–Whitney U test with n = 12 per group at power .80 and α = .05, the minimum detectable effect is approximately |r| = 0.62, rising to roughly 0.67–0.73 under Bonferroni–Holm adjustment. The two significant contrasts sit well above this threshold (|r| = 0.96 and 0.82); the secular self-regulation effect (|r| = 0.36) falls below it and is therefore not interpreted as equivalence.

*Note. U is the Mann–Whitney statistic for the pre-registered direction. Holm-adjusted p-values control the family-wise error rate across the five tests. |r| is the rank-biserial correlation. All values derive from the series-level lemma-pool frequencies tabulated in Appendix A.*
