# Supplementary material for: Partial substitution in the hidden curriculum of children’s media: a comparative corpus analysis of religion-oriented and general-educational Turkish public broadcasting
Source: Front Psychol. 2026 Aug 25;17:1887219. doi: 10.3389/fpsyg.2026.1887219 (PMC13549891; doi:10.3389/fpsyg.2026.1887219)
Supplement: Supplementary file 5 [file supplementary_file_5.docx]

Supplementary Methodological Appendix E

**Coding Scheme, Inter-Coder Reliability Protocol,**

**and Disagreement Resolution Procedure**

Companion document to §3.6 of the main manuscript

This appendix specifies the operational coding scheme that produced the inter-coder reliability statistic reported in §3.6 of the main manuscript (mean Cohen's κ = 0.86; range 0.78–0.94). It is written as a stand-alone codebook so that a third party can audit, replicate, or extend the annotation pipeline. The scheme is organised in seven sections: pool inventory, coder profile and training, units and materials, inclusion and exclusion rules, decision rules for ambiguous cases, KWIC verification protocol, and inter-coder reliability calculation including disagreement adjudication.

# 1. Pool Inventory and Operational Definitions

The annotation framework consists of twenty mutually informative pools organised into four groups: the five CASEL competencies (C1–C5), the ten Schwartz value categories (S1–S10), three self-regulation / religious-spiritual pools (RV, R1, R2), and two affect-lexicon pools (T1, E1). Pool membership is determined at the lemma level. Multi-pool membership is permitted under the conditions specified in §5.

| **ID** | **Pool** | **Operational core** | **Primary discrimination boundary** |
| --- | --- | --- | --- |
| C1 | CASEL: Self-awareness | Lexical reference to recognising one's own emotion, value, or thought-state | Excludes object-level emotion talk routed to C7 (general emotion). |
| C2 | CASEL: Self-management | Behavioural / impulse regulation in non-religious framing | Hardest boundary: with R2 (secular self-regulation). See §5. |
| C3 | CASEL: Social awareness | Empathy / perspective-taking lexemes | Boundary with S9 (benevolence): empathy ≠ active helping. |
| C4 | CASEL: Relationship skills | Cooperation, communication, conflict-resolution lexemes | Boundary with S9 (benevolence): act vs. disposition. |
| C5 | CASEL: Responsible decision-making | Ethical reasoning, consequence appraisal | Boundary with S8 (tradition) when moral norms are religiously grounded. |
| S1 | Schwartz: Self-direction | Independent thought, autonomy, exploration | Boundary with S2: curiosity-driven vs. arousal-driven. |
| S2 | Schwartz: Stimulation | Novelty, excitement, courage in face of risk | Boundary with S1 above and S3 (hedonism). |
| S3 | Schwartz: Hedonism | Sensory / immediate pleasure | Excludes long-horizon satisfaction routed to S4. |
| S4 | Schwartz: Achievement | Success, ability, attainment-by-effort | Boundary with S5: own-effort vs. control-of-others. |
| S5 | Schwartz: Power | Authority, status, control over others | Excludes self-mastery (routed to C2 / S4). |
| S6 | Schwartz: Security | Safety, health, trust, social order | Boundary with T1 (trust emotion): structural vs. affective. |
| S7 | Schwartz: Conformity | Obedience, rule-following, harmony with norms | Boundary with S8 when the norm is religious. |
| S8 | Schwartz: Tradition | Cultural / religious tradition, heritage, custom | Overlaps RV (religious vocabulary); double-coding allowed (see §3). |
| S9 | Schwartz: Benevolence | Care for in-group, kindness, loyalty | Boundary with S10: in-group vs. universal scope. |
| S10 | Schwartz: Universalism | Justice, equality, nature, peace at universal scope | Boundary with S9 (in-group) and C3 (perspective). |
| RV | Religious / spiritual vocabulary | Direct religious referents: Allah, peygamber, namaz, dua, oruç… | Highest-agreement pool; explicit Islamic-Turkish referents. |
| R1 | Religious self-regulation | Strategy lexemes embedded in religious frame (sabır, şükür, tevekkül, dua) | Requires KWIC verification of religious framing within ±5 tokens. |
| R2 | Secular self-regulation | Non-religious strategy lexemes (nefes, say-, sakinleş-, planla-, odaklan-) | Boundary with C2 (self-management): technique vs. disposition. |
| T1 | Trust emotion | Trust, certainty, hope, security as affect | Boundary with S6: affective vs. structural. |
| E1 | General emotion vocabulary | Emotion lexemes (mutlu, üzgün, korku, kızgın, gurur) | Catch-all for emotion words not consumed by C1 / T1. |

*Note. Lemma examples are illustrative; the full lemma list for each pool is provided in Appendix A of the main manuscript. The 'Primary discrimination boundary' column identifies the pool with which the focal pool is most easily confused; this is the boundary at which inter-coder disagreement is expected to concentrate (cf. §7).*

# 2. Coder Profile and Training

## 2.1. Coder profile

Two independent coders annotated the corpus. Both were specialists in language education holding doctoral degrees, one at full-professor rank, with published research on language education and learning. Both were native speakers of Turkish and held at least intermediate familiarity with the CASEL framework and Schwartz value theory through prior coursework or research experience. Neither coder was involved in formulating the research hypotheses, and neither was affiliated with the broadcasters under analysis. A third senior researcher (also a native Turkish speaker with doctoral training in educational psychology) served as adjudicator; the adjudicator did not participate in primary coding.

## 2.2. Training protocol

Training proceeded across four sequential phases:

1. **Theoretical familiarisation.** Both coders read Mahoney et al. (2018) on CASEL, Schwartz (1992, 2012) on the value circumplex, McCullough and Willoughby (2009) on religious self-regulation, and Bishop et al. (2004) on operational mindfulness. A two-hour calibration meeting reviewed the boundary distinctions tabulated in §1.
2. **Pilot calibration round.** A pilot lemma set of n = 120 candidates was extracted from a randomly chosen 10% sub-sample of episodes (balanced across channels). Both coders independently classified each lemma; agreement was computed at the end of the round.
3. **Disagreement-driven refinement.** Each pilot disagreement was reviewed jointly with the adjudicator. Resolutions were written into the working version of this codebook (Rules D-1 through D-3 in §5; Rules X-1 through X-3 in §4 originated in this phase). The pilot was repeated on a second n = 120 set until κ ≥ 0.80 was achieved on the pilot itself (achieved on the second pilot round).
4. **Main coding.** After calibration, the full candidate lemma set (n ≈ 2,180 after morphological consolidation) was coded. A 20% double-coded sub-sample (n = 4,360 lemma-pool decisions across the 20 pools) was reserved for the final reliability statistic. The 80% remainder was single-coded with random spot checks (5%) by the adjudicator.

# 3. Units of Analysis and Materials

## 3.1. Coding unit

The coding unit is the lemma. Each lemma decision is a binary in-pool / out-of-pool judgment per pool; a lemma may be in zero, one, or two pools (multi-pool conditions in §5). The reliability statistic is computed per pool over the double-coded sub-sample.

## 3.2. Materials

Coders worked from three artefacts simultaneously: (a) the concordance interface of a rule-based morphological analyzer set to a ±5 token window around the target lemma; (b) the lemma frequency report (raw and fpmw); and (c) the working codebook (this document). All decisions were recorded in a shared spreadsheet with one row per lemma and one column per pool.

# 4. Inclusion and Exclusion Rules

## 4.1. Inclusion rules (I-series)

- **I-1. Semantic-core match.** A lemma is included in a pool when its prototypical sense, as established by the TS Corpus (Aksan et al., 2012) primary entry, falls within the pool's operational core (column 3 of the §1 table).
- **I-2. KWIC majority rule.** Inclusion is confirmed when ≥ 70% of the lemma's KWIC occurrences (±5 tokens) instantiate the target sense in the source corpus. The 70% threshold was set during the pilot and held constant for the main coding.
- **I-3. Morphological expansion.** Productive Turkish derivational forms of an included lemma (e.g., –lI, –sIz, –lIk where the polarity does not reverse the sense) are admitted as members of the same pool. The lemmatiser already consolidates inflectional forms; derivational expansion is verified by hand.

## 4.2. Exclusion rules (X-series)

- **X-1. Negation reversal.** Lemma instances suffixed with –mA, –mAz, or derivational –sIzlIk that reverse the polarity of the target sense are excluded from frequency counts. Example: *sabırsız* (impatient) is not counted toward *sabır* under R1 / C2.
- **X-2. Off-target sense.** Instances in which the lemma does not instantiate the pool's target sense are excluded. Example: *şükür* used as a secular interjection ("şükür ki erken çıktık") is excluded from R1; whether the secular interjection enters E1 depends on the Rule D-2 routing.
- **X-3. Frozen idiom.** When ≥ 70% of occurrences appear within a fixed multi-word idiom that no longer indexes the literal sense (e.g., *son nefesine kadar* = "until the last gasp" used metaphorically for endurance), the lemma is excluded from the pool tied to the literal sense (R2) but may be admitted into an alternative pool if Rule D-1 applies.
- **X-4. Proper-noun residue.** Character names, series names, broadcaster identifiers, YouTube-platform terms, and series-specific noise ("inci", "hey") are excluded a priori. Religious proper nouns (Allah, peygamber, Kâbe, Zemzem) are retained as content markers under RV and S8 / R1 as appropriate.

# 5. Decision Rules for Ambiguous Cases

## 5.1. Rule D-1: Multi-pool candidacy

When two pools each satisfy Rule I-2 for the same lemma at ≥ 40% of independent KWIC instances, the lemma receives membership in both pools and its frequency is split in proportion to the KWIC distribution. Example:

*yardım* (help / aid) satisfies both S9 (benevolence) and C4 (relationship skills) in the corpus; it is split 60% S9 / 40% C4 on the basis of the KWIC sample.

## 5.2. Rule D-2: Affective vs. structural routing

Lemmas that index both an affective state (T1 / E1) and a structural condition (S6 / S5 / S2) are routed to the affective pool when the dominant KWIC sense is internal to the speaker, and to the structural pool when the dominant sense is external. Example:

*güven* (trust / confidence) used as "kendine güven" (trust yourself) routes to T1; used as "güvenli bir bölge" (a safe zone) routes to S6.

## 5.3. Rule D-3: Self-management vs. secular self-regulation (the hardest boundary)

This is the boundary at which the lowest pool-level κ values were observed (R2 = 0.81, C2 = 0.78), and so the rule is specified with particular care.

- If the lemma names a *specific technique* (breathing, counting, postponement, mindfulness, focused attention) → R2.
- If the lemma names a *dispositional or characterological state* (patience-as-trait, self-discipline, impulse-control-as-disposition) without specifying an enacted technique → C2.
- If the KWIC window contains an explicit religious anchor (Allah, Rab, ya Rabbi, dua, Kur'an, namaz) within the ±5 span → R1, overriding both C2 and R2.

## 5.4. Rule F-1: Religious framing detection

For the lemmas that participate in both R1 (religious self-regulation) and the secular alternatives (C2 or R2), operationally the strategy lexemes {sabır, şükür, tevekkül, dua, niyet, oruç, sabretmek, şükretmek, tevekkül etmek}, the religious framing test is applied. The test scans the ±5 KWIC window for the religious-anchor inventory listed under Rule D-3. Religious framing is confirmed if the anchor co-occurs in ≥ 50% of the lemma's KWIC instances; in that case all in-corpus occurrences of the lemma are routed to R1. Below the 50% threshold, the lemma is routed by literal sense (technique → R2; disposition → C2).

## 5.5. Rule D-4: In-group vs. universal scope (S9 vs. S10)

Benevolence (S9) is reserved for kindness directed at concrete, named, or proximal in-group members (family, friends, classmates, the umma when discursively bounded). Universalism (S10) is reserved for kindness or justice claims with explicitly universal scope ("tüm insanlar," "herkes için," "dünya barışı," "doğa"). If scope is ambiguous, the default is S9; KWIC verification is required for any S10 assignment.

# 6. KWIC Verification Protocol

Every pool assignment is supported by a KWIC sample of the lemma at ±5 tokens. The protocol is fixed at four steps:

5. Generate the full concordance for the target lemma , sorted alphabetically by left context to expose collocational regularities.
6. Inspect up to 50 randomly selected concordance lines (or all lines if total occurrences ≤ 50). Random selection uses a fixed-seed 'random sample' function with a fixed seed (=1729) recorded for replicability.
7. Classify each inspected KWIC instance as target-sense, off-target, negation-reversed, or idiomatic. Tally the proportions and apply Rules I-2, X-1, X-2, X-3.
8. Where Rules D-1 through D-4 or F-1 are triggered, record the precipitating KWIC line(s) in the decision spreadsheet so the adjudicator can audit the call.

The full coding decision flow is summarised below.

| **Step** | **Action** |
| --- | --- |
| 1 | Retrieve the candidate lemma from the working list. Confirm lemmatisation is correct against the morphological analyzer's output; if mis-lemmatised, flag as 'M' and skip. |
| 2 | Pull all KWIC concordance lines for the lemma at ±5 tokens. If n_occurrences < 3 across the merged corpus, mark as 'low-frequency' and exclude from reliability sample. |
| 3 | Apply the negation filter (X-1): if ≥ 50% of occurrences carry a Turkish negation suffix (–mA-, –mAz, –sIzlIk) and the polarity reverses the target sense, exclude the lemma. |
| 4 | Apply the idiom / metaphor filter (X-3): if ≥ 70% of occurrences are within a fixed multi-word expression that no longer indexes the literal sense, exclude. |
| 5 | Apply the primary-pool decision: match lemma to the pool whose operational core is satisfied by ≥ 70% of the KWIC sample. Record pool ID. |
| 6 | Apply the multi-pool rule (D-1): if a second pool is satisfied by ≥ 40% of independent KWIC instances, record secondary pool. Lemma frequency is split proportionally. |
| 7 | Apply religious-framing rule (F-1): for lemmas in {sabır, şükür, tevekkül, dua, oruç-related strategy verbs}, check the ±5 KWIC for a religious anchor (Allah, Rab, peygamber, Kur'an, namaz, ya Rabbi, vocative). If anchor present in ≥ 50% of occurrences, assign R1; otherwise C2 / R2 per literal sense. |
| 8 | Record final pool assignment(s) and flag any case requiring adjudication (entries marked 'D' or any disagreement with co-coder). |

# 7. Inter-Coder Reliability Calculation

## 7.1. Reliability statistic and rationale

Inter-coder agreement is calculated per pool as Cohen’s κ on the binary in-pool / out-of-pool decision. Cohen’s κ corrects observed agreement for chance under the assumption of independent coder judgments and is the field-standard statistic for two-coder categorical agreement (Cohen, 1960; Hallgren, 2012). It is used as the sole agreement measure in this study, reported per pool and as an unweighted mean across pools.

## 7.2. Sample structure

The 20% double-coded sub-sample yielded 4,360 lemma-pool decisions (218 lemmas × 20 pools, on average). Pool-level sample sizes vary because the candidate set for each pool depends on the lemma's semantic neighbourhood; pools with broader semantic territory (CASEL competencies, general emotion) drew larger candidate sets than pools with sharply bounded vocabulary (conformity, hedonism).

## 7.3. Pool-level results

Per-pool κ values are reported in Table E.1 below. Confidence intervals are computed via the standard normal approximation (Fleiss et al., 2003); for small pools where the approximation is unreliable, bootstrap CIs (10,000 resamples) were used and yielded substantively equivalent intervals.

**Table E.1**

*Pool-level inter-coder reliability statistics on the 20% double-coded sub-sample.*

| **Pool** | **Items double-coded** | **% agreement** | **Cohen's κ** | **95% CI** |
| --- | --- | --- | --- | --- |
| RV. Religious/spiritual vocabulary | 312 | 98.7% | 0.94 | [0.91, 0.97] |
| S8. Schwartz: Tradition | 264 | 98.1% | 0.93 | [0.89, 0.96] |
| S4. Schwartz: Achievement | 198 | 97.0% | 0.92 | [0.88, 0.96] |
| S5. Schwartz: Power | 142 | 96.5% | 0.90 | [0.85, 0.95] |
| S1. Schwartz: Self-direction | 224 | 96.9% | 0.89 | [0.85, 0.93] |
| S10. Schwartz: Universalism | 186 | 96.2% | 0.89 | [0.84, 0.94] |
| S2. Schwartz: Stimulation | 158 | 95.6% | 0.88 | [0.83, 0.93] |
| C5. CASEL: Responsible decision | 318 | 94.7% | 0.88 | [0.84, 0.92] |
| S6. Schwartz: Security | 192 | 94.8% | 0.87 | [0.82, 0.92] |
| S3. Schwartz: Hedonism | 134 | 94.0% | 0.87 | [0.81, 0.93] |
| R1. Religious self-regulation | 226 | 94.7% | 0.86 | [0.81, 0.91] |
| E1. General emotion vocabulary | 304 | 93.4% | 0.86 | [0.82, 0.90] |
| S7. Schwartz: Conformity | 96 | 93.8% | 0.85 | [0.78, 0.92] |
| T1. Trust emotion | 156 | 92.9% | 0.85 | [0.79, 0.91] |
| C1. CASEL: Self-awareness | 246 | 92.7% | 0.84 | [0.79, 0.89] |
| C4. CASEL: Relationship skills | 268 | 92.2% | 0.83 | [0.78, 0.88] |
| C3. CASEL: Social awareness | 222 | 91.4% | 0.82 | [0.77, 0.87] |
| R2. Secular self-regulation | 178 | 91.0% | 0.81 | [0.75, 0.87] |
| S9. Schwartz: Benevolence | 282 | 90.8% | 0.79 | [0.74, 0.84] |
| C2. CASEL: Self-management | 254 | 90.6% | 0.78 | [0.73, 0.83] |
| **Average across 20 pools (unweighted)** | **4,360 lemma decisions** | **94.0%** | **0.86** | **range 0.78–0.94** |

The unweighted mean of pool-level κ values is 0.86, and the observed range (0.78–0.94) corresponds exactly to the figures cited in §3.6 of the main manuscript. Following the Landis and Koch (1977) benchmarks, all pools fall in the 'substantial' (κ = 0.61–0.80) or 'almost perfect' (κ = 0.81–1.00) bands; no pool falls below the substantial threshold.

## 7.4. Pattern of disagreement

Disagreement concentrates predictably at the boundaries identified in §1, column 4. The two lowest κ values (C2 = 0.78, S9 = 0.79) sit at the C2 ↔ R2 and S9 ↔ C4 boundaries respectively. These are precisely the boundaries at which the operational distinctions are subtlest: enacted technique versus characterological disposition (C2 ↔ R2), and in-group disposition versus relational act (S9 ↔ C4). The highest κ values (RV = 0.94, S8 = 0.93) sit at pools anchored in explicit Islamic-Turkish lexis where the operational core is least ambiguous. This pattern provides convergent validity for the codebook: where the conceptual boundary is sharp, the coders agree; where it is operationally subtle, principled disagreement remains visible but is bounded by the decision rules.

# 8. Disagreement Adjudication

Any disagreement on the double-coded sample, plus any case marked 'D' by either coder during the single-coded portion, was referred to the adjudicator. The adjudication protocol is fixed:

9. The adjudicator reviewed the KWIC sample independently, without seeing the two coders' decisions.
10. The adjudicator applied the codebook rules and recorded a decision.
11. The two original decisions and the adjudicator's decision were then compared. Where the adjudicator's decision matched one of the original coders, that decision was final. Where the adjudicator's decision differed from both, a three-way discussion was convened to either (a) refine the relevant decision rule or (b) confirm the adjudicator's call by majority.
12. All rule refinements were dated and entered into a revision log appended to this codebook. Refinements made mid-coding were retroactively applied to all earlier decisions in the affected pool, and the affected portion of the double-coded sub-sample was re-scored.

Across the main coding, 412 lemma-pool decisions (≈ 9.4% of the double-coded sample) required adjudication. Of these, 387 (94%) were resolved by matching one of the original coders. The remaining 25 cases triggered three-way discussion; 18 led to rule refinements (the most consequential being the 50% threshold in Rule F-1, which was raised from the pilot's 40% on the basis of these cases), and 7 were settled by majority confirmation of the adjudicator's reading.

# 9. Worked Examples

The following table illustrates the decision procedure on ten representative lemma–KWIC pairs drawn from the corpus.

| **Lemma** | **KWIC excerpt (±5)** | **Coder A** | **Coder B** | **Adjudicated** |
| --- | --- | --- | --- | --- |
| *sabır* | "ya Rabbi sabır ver bana bir an önce iyileşeyim" | R1 | R1 | R1 (agreement) |
| *sabır* | "biraz sabırlı ol şişer elbet bu balon" | C2 | R2 | C2 (Rule D-3) |
| *şükür* | "Allah'a şükür sağlığım yerinde" | R1 | R1 | R1 (agreement) |
| *şükür* | "şükür ki erken ayrıldık trafikten kurtulduk" | E1 | excluded | excluded (Rule X-2) |
| *yardım* | "arkadaşına yardım etmek güzel bir davranıştır" | S9 | C4 | S9 + C4 (Rule D-1) |
| *nefes* | "derin nefes alıyoruz şimdi yavaşça veriyoruz" | R2 | R2 | R2 (agreement) |
| *nefes* | "son nefesine kadar koştu ve çizgiyi geçti" | S4 | excluded | excluded (Rule X-3, idiom) |
| *dürüst* | "dürüst olmak her zaman doğrudur" | C5 | C5 | C5 (agreement) |
| *güven* | "kendine güven, başarabilirsin" | T1 | C1 | T1 (Rule D-2) |
| *tevekkül* | "Allah'a tevekkül et evladım" | R1 | R1 | R1 (agreement) |

*Note. KWIC excerpts are reproduced from the main manuscript (§4.3) and from the corpus directly. Pool codes correspond to the inventory in §1.*

# 10. Reporting Conventions

The main-text reporting follows the conventions established here:

- Per-pool κ values are reported in the supplementary table above. Only the summary statistic (mean κ = 0.86, range 0.78–0.94) appears in §3.6 of the main text.
- Disagreement-rate per pool is available on request but is not reported in the main text; the κ value already incorporates this information.
- Confidence intervals on κ are not reported in the main text but are included here for transparency. No pool's lower 95% CI bound falls below 0.73.
- Where multi-pool memberships are coded under Rule D-1, the lemma is counted once in each receiving pool with frequency split per the KWIC distribution. This split is preserved through the Simple Maths, G², and DP computations reported in §4 of the main manuscript.

# 11. Limitations of the Coding Scheme

Three limitations of the codebook are acknowledged here in full. First, the 70% KWIC majority threshold (Rule I-2) is a calibration decision rather than a derived parameter; sensitivity analyses at 60% and 80% (not reported in the main text) shifted pool-level frequencies by less than 4% in aggregate but altered the boundary-crossings at C2 ↔ R2 modestly. Second, multi-pool coding under Rule D-1 introduces a methodological choice that is not field-standard; the alternative single-pool-only scheme was tested in pilot 2 and produced systematically lower κ values (mean 0.79) by forcing arbitrary disambiguation, which justifies the multi-pool decision. Third, the codebook is calibrated on Turkish corpus material and on the Islamic-Turkish religious referent set; adaptation to other languages or religious traditions would require recalibration of the religious-anchor inventory in Rule D-3 and Rule F-1.

# References cited in this appendix

Aksan, Y., Aksan, M., Koltuksuz, A., et al. (2012). Construction of the Turkish National Corpus (TNC). In Proceedings of the Eighth International Conference on Language Resources and Evaluation (LREC'12).

Bishop, S. R., Lau, M., Shapiro, S., et al. (2004). Mindfulness: A proposed operational definition. Clinical Psychology: Science and Practice, 11, 230–241.

Cohen, J. (1960). A coefficient of agreement for nominal scales. Educational and Psychological Measurement, 20(1), 37–46.

Fleiss, J. L., Levin, B., & Paik, M. C. (2003). Statistical methods for rates and proportions (3rd ed.). Wiley.

Hallgren, K. A. (2012). Computing inter-rater reliability for observational data: An overview and tutorial. Tutorials in Quantitative Methods for Psychology, 8(1), 23–34.

Landis, J. R., & Koch, G. G. (1977). The measurement of observer agreement for categorical data. Biometrics, 33(1), 159–174.

Mahoney, J. L., Durlak, J. A., & Weissberg, R. P. (2018). An update on social and emotional learning outcome research. Phi Delta Kappan, 100(4), 18–23.

McCullough, M. E., & Willoughby, B. L. B. (2009). Religion, self-regulation, and self-control. Psychological Bulletin, 135(1), 69–93.

Schwartz, S. H. (1992). Universals in the content and structure of values. Advances in Experimental Social Psychology, 25, 1–65.

Schwartz, S. H. (2012). An overview of the Schwartz theory of basic values. Online Readings in Psychology and Culture, 2(1).
